# *Savor‐Aging*: The Art of Savoring Positive Emotions in Older Adulthood—A Randomized Controlled Trial

**DOI:** 10.1111/aphw.70184

**Published:** 2026-07-04

**Authors:** Elisa Pancini, Giulia Cremaschi, Marta Degani, Elisa Cisotto, Daniela Villani

**Affiliations:** ^1^ Research Center in Communication Psychology (PsiCom), Department of Psychology Università Cattolica del Sacro Cuore Milan Italy; ^2^ Department of Psychology Università Cattolica del Sacro Cuore Milan Italy; ^3^ Department of Statistical Sciences Università Cattolica del Sacro Cuore Milan Italy

**Keywords:** depression, older adults, online intervention, positive emotions, savoring

## Abstract

Savoring has been identified as a promising strategy to foster emotional and psychological well‐being and to reduce depression in elders. However, research on online savoring interventions for this population remains limited. This registered RCT aimed to examine the effectiveness of a 3‐week online intervention (six activities), Savor‐Aging, in promoting subjective and psychological well‐being and reducing depressive symptoms. Seventy‐six healthy older adults (*M* = 69.9, *SD* = 4.8) were randomly assigned to a savoring group or a positive emotion (PE) group. Life satisfaction, positive and negative affect, flourishing, and depression were assessed at baseline (T0), post‐intervention (T1), and 1‐month follow‐up (T2) to test psychological changes. Longitudinal quantitative data were analyzed using linear mixed‐effects models, whereas qualitative feedback was thematically analyzed. Significant overall time effects emerged for negative affect and depressive symptoms. Although most time × group interactions were not significant, the savoring condition showed more consistent improvements over time, including reductions in negative affect and depressive symptoms that were maintained at follow‐up. Significant time effects emerged for flourishing only in the savoring group immediately after the intervention. Participants described the savoring intervention as pleasant, engaging, and meaningful. Overall, Savor‐Aging appears to be a feasible and effective self‐help intervention to support emotional well‐being and reduce depression in older adults, offering an accessible approach to positive aging.

## INTRODUCTION

### Positive mental health and well‐being in older adults

Aging is a complex process that can be characterized by several factors, including reduced functional and physical abilities, a progressive loss of independence, a shrinking circle of friends, and an increase in experiences of bereavement (Baird et al., 2010). Although some studies suggest that well‐being follows a U‐shaped trajectory across the lifespan, with relatively higher levels in older age compared to midlife (Blanchflower & Oswald, 2008), this pattern is not universal. Indeed, the challenges associated with aging may, for some individuals, contribute to a gradual decline in well‐being, often accompanied by changes in emotional experience (Ramírez et al., 2014; Stone et al., 2020; Tibubos et al., 2025). For example, in Italy, the lowest scores of mental well‐being are observed in individuals over the age of 75, with this trend being more pronounced among women (Istat, 2024). This reduction in well‐being, combined with the previously mentioned factors, may increase the risk of developing depressive symptoms such as persistent sadness and hopelessness (Sözeri‐Varma, 2012). For instance, the World Health Organization (WHO) has reported that approximately 14% of adults aged 60 years and older globally suffer from a mental disorder, such as depression (WHO, 2023, 2025).

These widespread challenges in the older population have led researchers to identify methods and strategies to promote both subjective well‐being and psychological well‐being. Specifically, subjective well‐being refers to how people experience and evaluate their lives. It includes aspects such as life satisfaction, positive emotions, and low negative emotions (Diener et al., 1985). Psychological well‐being, on the other hand, concerns the realization of one's full potential and personal growth (Ryff, 1989). A strategy that can be employed to promote these dimensions is savoring (Bryant & Veroff, 2007). Savoring emerges as a key tool for amplifying positive emotions and promoting mental well‐being. This strategy is related to a theoretical framework developed by Fredrickson: the *Broaden‐and‐Build Theory of Positive Emotions*. This theory posits that positive emotions generate beneficial effects in both the short and long term, in contrast to negative emotions (Fredrickson, 1998). In terms of short‐term benefits, positive emotions broaden individuals' attentional scope, enhancing their ability to perceive the overall view and increasing awareness of the surrounding environment. Moreover, positive emotions can act as facilitators for the expansion of one's thought–action repertoires, for example, by stimulating the exploration of new activities. In addition, the presence of positive emotions fosters more open and prosocial mindsets, increasing individuals' willingness to engage in socially supportive behaviors (van Cappellen & Fredrickson, 2025). Furthermore, according to this theory, positive emotions contribute to building enduring personal resources (Fredrickson, 2004). In the long term, the presence of positive emotions facilitates the development of lasting relationships, improved physical and mental health, and greater resilience (Fredrickson et al., 2021; Gloria & Steinhardt, 2016; van Cappellen & Fredrickson, 2025). As such, positive emotions can impact the well‐being of older adults through both short‐ and long‐term benefits, encouraging engagement in a broader range of thoughts and actions and promoting better adaptation and healthier lifestyles (Salovey et al., 2000).

Savoring can be defined as “the ability to attend to, appreciate, and enhance positive experiences in one's life” (Bryant & Veroff, 2007, p. xi), and it enables individuals to broaden and prolong the benefits derived from positive experiences and emotions (Quoidbach et al., 2010; Smith & Bryant, 2019). Through this process, individuals intentionally engage in thoughts and behaviors aimed at regulating their positive emotions, thereby increasing their intensity, duration, and frequency (Bryant et al., 2011). Savoring can also be promoted through behaviors and thoughts that intensify positive experiences (Bryant & Veroff, 2007). Examples include sharing the experience with others, creating positive memories, expressing joy through behavior, becoming absorbed in the moment, and self‐congratulation. Savoring can have three temporal orientations, including *positive reminiscence*, recalling a past positive event allows individuals to re‐experience the original emotion and possibly elicit new positive emotions (e.g., remembering a vacation); *savoring the present moment*, focusing on and amplifying the emotions experienced during a current activity (e.g., during a walk in the park, paying attention to the sounds, the warmth of the sun, bodily sensations, and related emotional states); and *positive anticipation*, appreciating in the present the positive emotions elicited by the expectation of a future event (e.g., looking forward to seeing a friend after a long time).

Several studies have examined the relationships between savoring and various well‐being indicators in the older adult population. Specifically, some authors have shown that higher savoring capacities in older adults predict greater happiness, life satisfaction, and perceived control, while also serving as a protective factor against depressive symptoms (Bryant, 2003; Ramsey & Gentzler, 2014; Smith & Hollinger‐Smith, 2015; Stephens et al., 2025). Through savoring, older adults can capitalize on positive emotions and improve their psychological well‐being (Jiao et al., 2021; Salces‐Cubero et al., 2019; Smith & Hanni, 2019). In particular, savoring the moment has been positively associated with higher quality of life, subjective and psychological well‐being, and positive affect (Wilson et al., 2025). Smith and Bryant (2016) corroborated these findings, demonstrating that older adults with stronger savoring abilities report higher life satisfaction regardless of their health status. Additionally, Wallimann et al. (2024) found that daily time spent savoring moderates the relationship between daily solitude and several negative outcomes, including depressive symptoms, loneliness, and somatic complaints. In recent years, the possibility of enhancing savoring through targeted strategies has led to the development of dedicated psychological interventions.

### In‐person and online savoring interventions in older adults

In light of these encouraging findings from the literature, it is worthwhile to explore the potential of interventions aimed at enhancing and supporting savoring abilities and the effects these may have on the well‐being of the older adult population. Up to now, most of the studies exploring the potential of savoring interventions have been focused on the adult and young adult populations (Villani et al., 2023). In fact, a meta‐analysis reveals that most of the savoring interventions yielding positive outcomes on affect and happiness were conducted on participants whose age ranged from 18 to 43 years (Smith et al., 2014). However, such interventions may be particularly valuable for older adults, as they align with age‐related changes such as enhanced emotional regulation capacities (Urry & Gross, 2010) and a greater tendency to prioritize emotionally meaningful goals due to increased awareness of life's limited duration (e.g., Carstensen & Charles, 1998; Charles & Carstensen, 2009). Consequently, some studies (Ho et al., 2014; Salces‐Cubero et al., 2019; Salces‐Cubero et al., 2025; Smith & Bryant, 2019) suggest that savoring‐based interventions could be promising tools for promoting older adults' psychological and emotional well‐being.

Up to now, savoring has been included in a general positive psychology intervention with a pre‐test/post‐test design, where older adults engaged in a savoring session of mindfully enjoying a cup of tea, followed by a group discussion and reflection on the experience (Ho et al., 2014). The results from the entire intervention highlight an interesting impact on depressive symptoms and on life satisfaction, gratitude, and happiness. On the other hand, a few specific savoring interventions have been developed and tested recently. Salces‐Cubero et al. (2019) tested a savoring intervention with a sample of 124 older adults aged 60 or over. The intervention consisted of four 70‐min presence meetings and was compared with a gratitude‐based intervention and an optimism‐based intervention. Participants in the savoring condition experienced increases in life satisfaction, positive affect, subjective happiness, and resilience, as well as reductions in negative affect and depressive symptoms. Additionally, Smith and Bryant (2019) proposed in their study a one‐shot manipulation task, which included a savoring aging reflection for the savoring group, a negative aging reflection for the control group, and no activity for the passive control group, in order to measure the effects on positive and negative perceptions of aging, satisfaction with life, and anxiety. Reflecting upon life lessons acquired over the course of one's lifetime led to life satisfaction and hope (Bryant et al., 2021), with positive affective states induced by savoring serving as mediators of these effects (Smith & Bryant, 2019). The savoring group reported greater life satisfaction compared to the other groups.

Savoring can be facilitated through exercises involving personal reflection and mindful attention to the sensations experienced while engaging in individual and social daily activities. These exercises can now be easily guided through online programs that provide access to digital texts, as well as audio or video recordings (Villani et al., 2023). In addition, the older adult population is more web‐friendly than ever, as shown by the data that 69.3% of 4492 older adults aged over 50 use the internet and e‐mail every day (Stockwell et al., 2021). Thus, in recent years, some online savoring interventions have been implemented, showing promising results. However, the paucity of studies currently available prevents a comprehensive understanding of the effectiveness of online savoring interventions with the elderly population.

Smith and Hanni (2019) conducted a 1‐week remote savoring intervention among older adults aged 60 or over. Specifically, all participants were asked to engage in and savor a positive activity for 5 min each morning and evening over the course of 1 week, the participants had received the instruction via email. Participants increased their resilience and happiness, as well as decreased depressive symptoms. These beneficial outcomes were observed among those who completed the activity for at least 6 out of 7 days, whereas no significant effects were found for participants who completed the activity on five or fewer days. According to the authors, savoring interventions may enhance the salience of positive experiences and, consequently, influence the encoding and memory storage of such events. Similar related effects on memory and recall capacities have also been documented in younger populations (Colombo et al., 2024). Recently, another web‐based intervention has been focused on relational savoring, a particular form of savoring focused on deepening and enjoying positive emotions generated through interpersonal relationships. The 3‐week pilot intervention has shown promising results in terms of savoring beliefs and the regulation of both positive and negative emotions (Pancini et al., 2023). Despite these encouraging preliminary findings, several critical gaps remain in the current literature. First, many existing studies rely on “one‐shot” manipulations (e.g., Smith & Bryant, 2019) or very brief activities (e.g., Ho et al., 2014). Moreover, even when remote interventions are conducted, they frequently have restricted durations; for example, the research by Smith and Hanni (2019) was confined to a single week. There is a clear need to explore more structured interventions to determine if extended practice leads to more robust benefits. Additionally, a significant methodological limitation in research involving the elderly is the lack of active control groups. Many studies either utilize only an experimental group (e.g., Friedman et al., 2017; Smith & Hanni, 2019) or a passive, untreated control group (e.g., Smith & Bryant, 2019), making it difficult to isolate the specific effects of savoring from general engagement in positive activities or placebo effects. Finally, the absence of follow‐up assessments in many existing protocols prevents a comprehensive understanding of the long‐term sustainability of these digital interventions.

### The present study

Overall, correlational evidence has consistently linked savoring abilities to higher levels of well‐being, whereas intervention studies have further demonstrated the potential of savoring‐based interventions to enhance well‐being and reduce depressive symptoms. Despite the observed benefits among older adults, there is a lack of research specifically targeting this population, particularly in the context of online savoring interventions.

To address this gap, we conducted a randomized controlled trial (RCT) to examine the effectiveness of a three‐week savoring intervention named *Savor‐Aging* in promoting subjective and psychological well‐being and reducing depression in a sample of older adults compared with an active control group. Specifically, outcomes were assessed from baseline (T0) to post‐intervention (T1) and at the 1‐month follow‐up (T2).

Based on the theoretical framework outlined above, we formulated the following hypotheses:

Hypothesis 1 Subjective well‐being would increase more in the savoring group than in the active control group, which participated in a positive emotion intervention (PE group). More precisely, we expected the savoring group to report higher levels of life satisfaction and positive emotions and lower levels of negative emotions compared to the PE group. The inclusion of an active PE group was intended to control for the general benefits associated with the knowledge and potential outcomes of positive emotions, in line with the *Broaden‐and‐Build Theory of Positive Emotions* (Fredrickson, 1998). Accordingly, the PE intervention was designed to elicit and increase the frequency of positive emotions, whereas the savoring intervention specifically targeted the regulation of positive emotions. In particular, savoring involves the intentional processes of attending to, amplifying, and prolonging positive emotional experiences across temporal frames (past, present, and future). Thus, rather than merely generating positive affect, savoring emphasizes metacognitive awareness and active engagement with positive experiences.

Hypothesis 2 Psychological well‐being would increase more in the savoring group than in the PE group. We expected these differences because savoring interventions not only elicit positive emotions but also foster greater awareness, appreciation, and prolonged engagement with positive experiences. This reflective and amplifying process is theorized to strengthen emotional regulation and build enduring psychological resources over time (Bryant & Veroff, 2007; Fredrickson, 1998).

Hypothesis 3 As a secondary outcome, depression would decrease more in the savoring group than in the PE group. Savoring is expected to reduce depressive symptoms by shifting attention away from negative thoughts and promoting the ability to notice and reappraise everyday positive experiences. Consequently, this process may help to build psychological resources that buffer against depressive states (Bryant & Veroff, 2007; Fredrickson, 1998).

Overall, by implementing a structured 3‐week intervention, this study aims to overcome the limitations of brief or one‐shot interventions. Crucially, the inclusion of an active control group and a 1‐month follow‐up (T2) allows for a more rigorous evaluation of whether savoring provides incremental benefits beyond general positive emotion induction and whether these improvements persist over time. Finally, we aimed to explore participants' evaluation of the interaction with the digital platform, their perceptions about the overall usefulness of the intervention and about the usefulness and pleasantness of the proposed exercises, the emotions they experienced during the exercises, and the content of the personal experiences they savored during the intervention.

## METHODS

### Study design and participants

This RCT had a between‐subjects design and included three different assessment moments: the initial baseline assessment (T0), the post‐intervention assessment (T1), and the 1‐month follow‐up (T2). Sample size calculations were performed using G*Power 3.1.9.7 (Kang, 2021). The calculation was based on a repeated‐measures ANOVA, specifically targeting the within–between interaction. The Type I error rate was set at 5% (*α* = .05), assuming a medium effect size (*f* = 0.25) for the primary outcomes. To achieve 95% power with two groups and three repeated measurements, a correlation of 0.5 among repeated measures and a non‐sphericity correction factor (*ɛ*) of 1.0 were assumed. Based on these parameters, a minimum of 44 participants were required. Due to the estimated difficulty of engaging older adults interested in the intervention and able to use the internet, few Universities of the Third Age in Northern Italy (Lombardy and Emilia‐Romagna regions) were contacted to propose the intervention to their members. They sent the study invitation via email, and 125 older adults expressed their interest in participating. The inclusion criteria were age above 60 years, a score of 26 or more on the Itel‐MMSE questionnaire administered during the telephone interview, fluency in the Italian language, and having Internet access.

**FIGURE 1:**
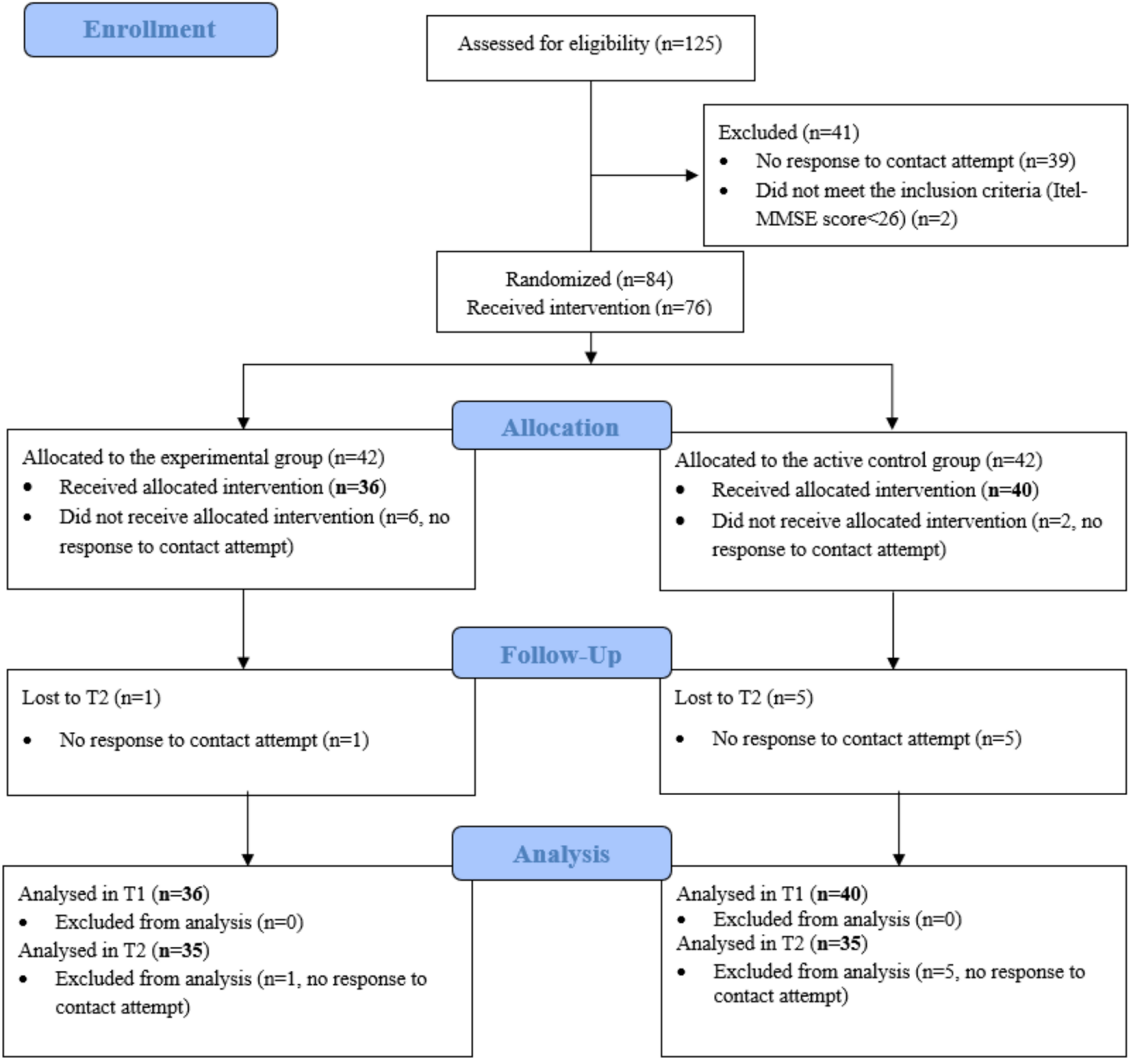
CONSORT flow diagram.

However, when contacted to formally begin the intervention, 39 participants did not respond or declined to proceed (Figure 1). In addition, two participants did not meet the inclusion criteria, as they scored below 26 on the Itel‐MMSE and were therefore excluded from the study. This attrition rate was in line with other similar studies on the same population (Bouwman et al., 2019). Therefore, 84 participants were randomly assigned by the researchers to the savoring and PE groups using block randomization. Each group was labeled as A or B (A = intervention, B = control; block size = 4). The randomization list was generated using free online software (Research Randomizer 4.0). The data were subsequently screened for outliers, and none were detected. Six participants of the savoring group and two participants of the PE group were excluded from the analysis because they stopped replying to researchers' e‐mails and did not complete the intervention activities and the baseline (T0) questionnaires. As a result, the final sample consisted of 76 participants, specifically 36 in the savoring condition (*M_age* = 70.20, *SD* = 5.19) and 40 in the PE condition (*M_age* = 69.60, *SD* = 4.50) (Figure 1). This study was approved by the Ethical Committee for Research in Psychology of the Department of Psychology of Università Cattolica del Sacro Cuore of Milan (Protocol Number: 128/24) and the RCT was prospectively registered on ClinicalTrials.gov (NCT06842628). Participants did not receive any financial or other compensation for their involvement in the study.

### Procedure

This study was conducted from January to June 2025. People interested in participating in the study, including those who had received direct communication, heard about it through contacts or through the Universities of the Third Age to which they belonged, contacted the researchers by e‐mail. Each older adult received, via e‐mail, a link containing the information sheet and informed consent form, along with a proposal for a telephone appointment with a researcher for the administration of the Itel‐MMSE, aimed at assessing the inclusion and exclusion criteria of the participants. At the end of the telephone call, the participants who met the inclusion and exclusion criteria were randomly assigned to the savoring group and the PE group and were subsequently informed by email about the start of the training. The savoring group followed the savoring intervention, with activities aimed at engaging participants in noticing, intensifying, and prolonging positive experiences (e.g., guided reflection, attention to sensory and emotional aspects, and personal meaning‐making). In contrast, the active control group followed the PE intervention focused on increasing understanding of positive emotions and eliciting them through structured activities (e.g., psychoeducation and emotion induction), without explicitly training participants in the regulatory processes' characteristic of savoring (i.e., amplification and prolongation of positive experiences). Both savoring and PE groups completed a 3‐week training with two activities per week. In addition, both groups were administered the same questionnaires (T0–T1–T2) in the same time frame and completed the short questionnaires designed to investigate the perceived enjoyment and usefulness of each activity. Each exercise took about 10 min to complete, and participants were given the opportunity to access the exercise in either recorded audio or written text format to provide participants the chance to choose their favorite modality.

**FIGURE 2:**
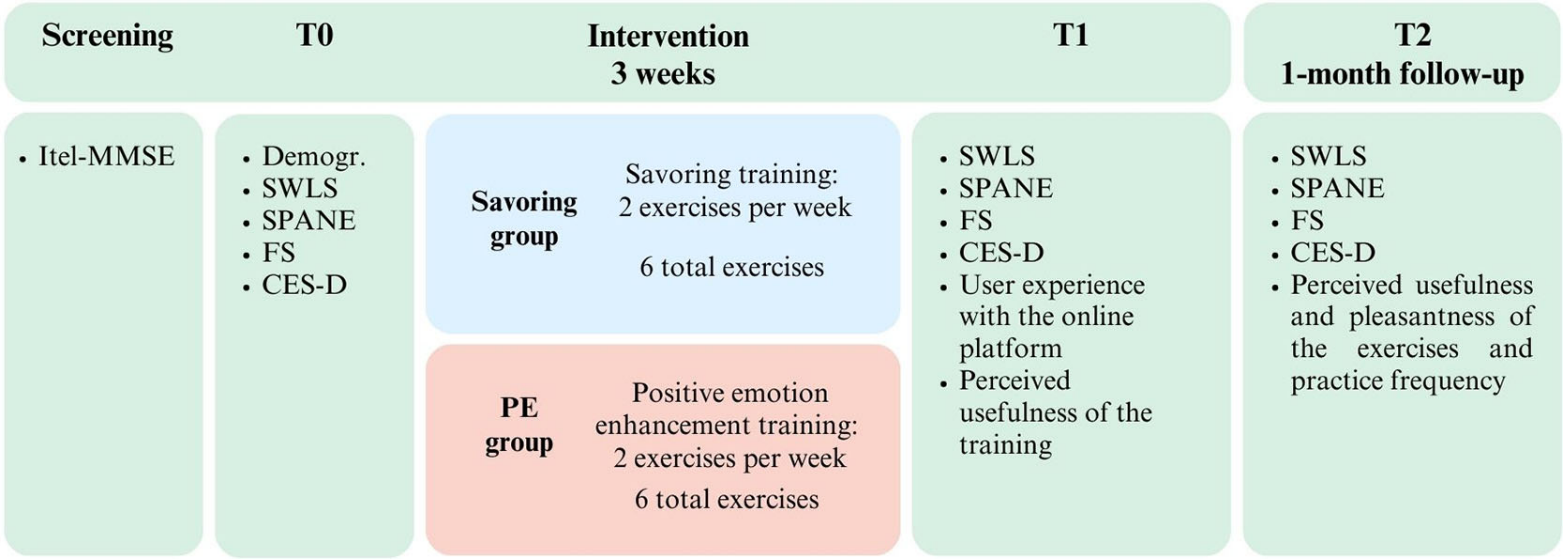
Study procedure.

**FIGURE 3:**
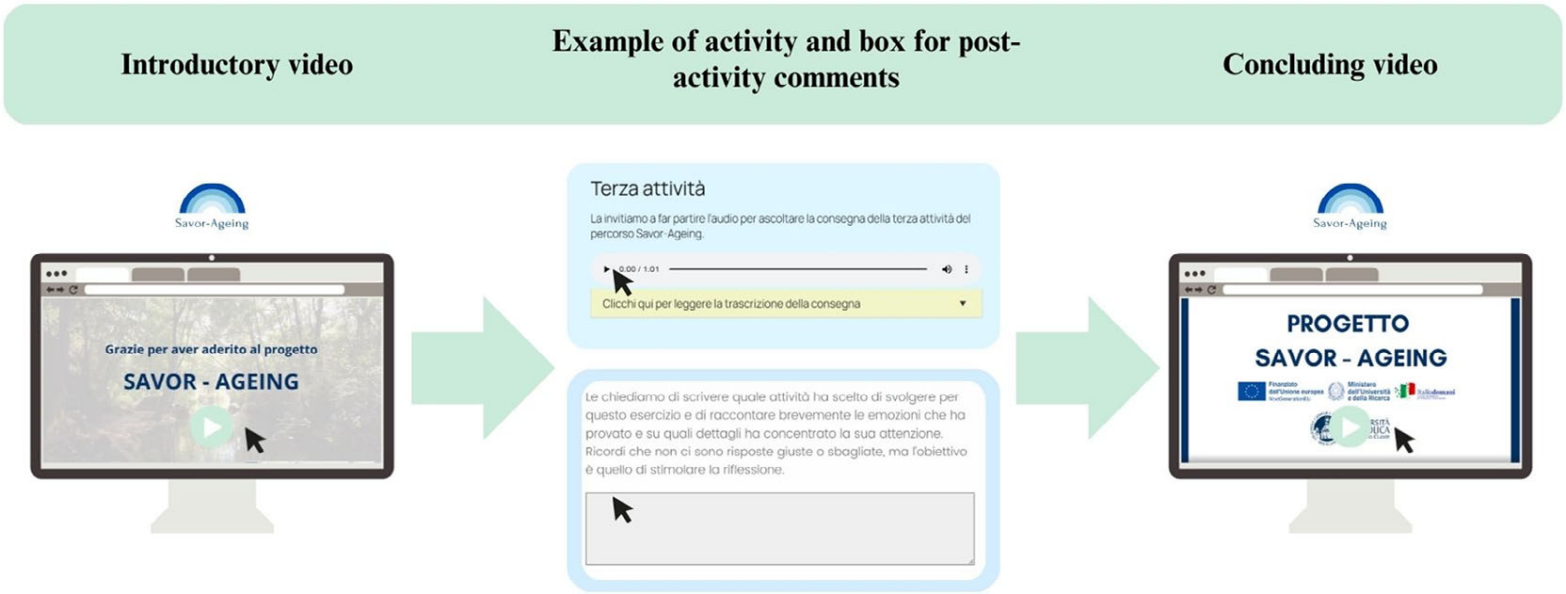
Intervention phases.

Once they were assigned to the groups, each participant received a Qualtrics link via e‐mail for filling out the T0 questionnaires. After completing the baseline assessment, they were given access to the website by sending a link and personalized credentials. After accessing the website, participants were invited to watch an introductory video that presented the research team, outlined the objectives of the training, and explained how the activities were organized over the 3 weeks, as well as the general structure of each session (Figure 2). At the end of each exercise, participants were invited to write down their reflections and personal savoring experiences (Figure 3). At the end of the training, each participant was invited to watch a concluding video and complete the T1 questionnaires. The video reviewed the various activities carried out during the training and encouraged participants to continue practicing the learned exercises in their daily lives. One month after the end of the intervention, the participants were contacted again by e‐mail for the last administration of the final T2 questionnaire (Figure 2). At the end of the training and after the compilation of the T2 questionnaires, both groups were given free access to the website for a whole year and were given two supplementary savoring exercises (described in Table 1). This opportunity was presented as a gift for both groups and allowed the PE group to experience the savoring activities included in the savoring intervention. Throughout the intervention, researchers were available to participants via email and phone, with all contact details displayed on the platform. Availability was communicated at multiple moments: in the informed consent form, during the Itel‐MMSE administration calls, in the introductory video, and in reminder emails. Participants were also informed of their right to withdraw at any time, for any reason, and without any consequences. No crisis episodes or adverse events occurred. Participants occasionally contacted the research team with questions about specific exercises or technical aspects of the platform; all queries were handled promptly, and no difficulties remained unresolved.

**TABLE 1:** Description of the savoring group's activities.

Week	Themes	Purpose	Brief description
1—focus on the past	Positive reminiscence	Enhancing awareness and savoring a positive past event, which may or may not be associated with another person. Furthermore, the objective is also to promote the process of immersion and the revival of positive emotions.	In this activity, participants are invited to recall a positive event from their past and focus on the positive emotions and physical sensations they experience through reminiscence. This activity was inspired by and adapted from a study by Villani et al. (2023). In the previous study, participants were instructed to recall a memory linked to someone. The present study allowed participants to decide whether they would like to recall a memory connected to someone or not.
1—focus on the past	Life lessons	Improving life satisfaction and positive attitudes to aging.	Participants are invited to choose a priceless life lesson they have learned during their life and to reflect on how their life without it could be. The activity also asks participants to focus on the emotions and physical sensations they feel during the reflection. This activity was inspired by and adapted from the studies of Smith and Bryant (2019) and Bryant et al. (2021). In these studies, the purpose of the activity was to reflect on a life lesson. The present study adds a reflection on positive emotions to improve savoring.
2—focus on the present	Absorption during an activity	Fostering focus on the present, amplifying perceptual experience, and increasing awareness of their positive emotions.	Participants are invited to perform an activity that they particularly enjoyed and to pay attention to the perceptual experience related to positive emotions. This activity was already used by Villani et al. (2023).
2—focus on the present	Gratitude letter	To make participants understand the importance of expressing gratitude toward loved ones and to be aware of the positive effect that this action could have on their day and their positive emotions.	Participants are invited to write a gratitude letter to a loved one and have the option of delivering it to that person. During the activity, participants are asked to focus on the positive emotions they felt while writing and delivering the letter. This activity was inspired by and adapted from a study by Heintzelman et al. (2020), in which the purpose of the activity was to improve gratitude only. The present study adds a reflection on positive emotions to improve savoring.
3—focus on the future	Anticipation of positive events	Raising awareness of the positive emotions that can be experienced by thinking about and visualizing positive future events.	Participants are asked to visualize positive future events that could happen in the next few hours, days, or weeks and to focus on the positive emotions they experience during the activity. This activity was already used by Villani et al. (2023).
3—focus on the future	Making a kind gesture	Stimulating positive emotions in the present by anticipating a kind gesture performed for a loved one.	Participants are invited to imagine performing a kind gesture for a loved one, focusing on the positive emotions they experience. They are also encouraged to perform the kind gesture while focusing on the positive emotions they feel doing that. This activity was inspired by and adapted from a study by Cohn et al. (2014), in which participants were asked to perform an act of kindness toward someone else and to record this act in a kindness diary. In the present study, participants are asked to imagine performing an act of kindness and to reflect on the emotions and sensations they experience.
Bonus Activity 1	Gratitude daily diary	To make participants reflect on the positive aspects in life that they are grateful for. They should also focus on the positive emotions experienced while doing that.	Participants are invited to take a moment each day to reflect on the things and people they are grateful for and to pay attention to any positive emotions or sensations they experience. This activity was inspired by and adapted from a study by Cohn et al. (2014), in which the purpose was to focus on positive daily events. In the present study, participants are asked to focus every day on what they are grateful for in their entire life.
Bonus Activity 2	List of positive events	Raising awareness of the positive events and successes that people have experienced in their lives. It also aims to improve the ability to savor these events and relive the positive emotions associated with those memories.	Participants are asked to make a list of three positive, pleasant, or joyful events they have experienced in their life, focusing on the associated positive emotions. This activity was already used by Smith and Hanni (2019).

### Measures

Detailed validity indicators, including factor loadings and model fit indices, were reported in Appendix S2.

### Inclusion criteria screening

In order to control the reliability of the questionnaire responses, all the participants were interviewed by telephone to assess the global cognitive functioning with the Itel‐MMSE.

#### Itel‐Mini Mental State Examination (Itel‐MMSE)

This scale was designed by Metitieri et al. (2001) to evaluate global cognitive functioning in the participants. It is administered by telephone, and it consists of six items about orientation, memory, calculation, attention, naming, and repetition, and it was used as an exclusion criterion. The score range is from 0 (maximum cognitive impairment) to 22 (no cognitive impairment). This score could be converted into the original MMSE score (Folstein et al., 1975; Italian version: Measso et al., 1993; Magni et al., 1996), whose range is from 0 (maximum cognitive impairment) to 30 (no cognitive impairment). In this study, this questionnaire was administered by telephone before the administration of the T0 questionnaire, and the score was used to determine the involvement of participants' responses. Any participants scoring below 26 would have been excluded from the study sample; two participants met this criterion and were therefore excluded. However, they were still granted access to the savoring intervention activities outside the research protocol.

### Primary outcome measures

The two groups completed the following online questionnaires on Qualtrics.

#### Satisfaction With Life Scale (SWLS)

The Satisfaction With Life Scale (SWLS), designed by Diener et al. (1985) (Italian version: di Fabio & Busoni, 2009), aims to investigate the cognitive dimension of subjective well‐being and consists of five items that can be answered through a seven‐step Likert scale ranging from 1 *strongly disagree* to 7 *strongly agree*. This questionnaire was administered at T0 (*α* = .90 and *⍵* = .91), T1 (*α* = .87 and *⍵* = .89), and T2 (*α* = .90 and *⍵* = .91).

#### Scale of Positive and Negative Experiences (SPANE)

This self‐report scale consists of 12 items and was developed by Diener et al. (2010) (Italian version: Corno et al., 2016) to measure the affective dimension of subjective well‐being. It is composed of two sub‐scales: The first refers to positive affect (SPANE‐P, at T0 *α* = .91 and *⍵* = 0.91; at T1 *α* = .91 and *⍵* = .91; at T2 *α* = .92 and *⍵* = .92) (six items: positive, good, pleasant, happy, joyful, and satisfied), and the second to negative affect (SPANE‐N, at T0 *α* = .82 and *⍵* = .84; at T1 *α* = .82 and *⍵* = .84; at T2 *α* = .76 and *⍵* = .78) (six items: negative, bad, unpleasant, sad, fearful, and angry). It can be answered through a five‐step Likert scale where 1 means *very rarely or never* and 5 means *very often or always*. This questionnaire was administered at T0–T2.

#### Flourishing Scale (FS)

The Flourishing Scale (FS), developed by Diener et al. (2010) (Italian version: di Fabio, 2016), aims to investigate the elements that make up psychological well‐being, including relationships, self‐esteem, life purpose, and optimism. It consists of eight items, each positively worded, and responses are measured on a seven‐step Likert scale, where 1 means *strongly disagree* and 7 means *strongly agree*. The total score is calculated by adding up the individual scores; the range is from 8 to 56. This questionnaire was administered (*α* = .89 and *⍵* = .90), T1 (*α* = .83 and *⍵* = .84), and T2 (*α* = .83 and *⍵* = .84).

### Secondary outcome measures

#### Center for Epidemiologic Studies‐Depression Scale (CES‐D)

Developed by Radloff (1977), the Center for Epidemiologic Studies‐Depression Scale (CES‐D) is a 20‐item self‐report scale used as a screening tool for depression in the elderly. For the purpose of this study, the 10‐item short version, validated by Andresen et al. (1994), was used. Participants were asked to indicate on a 5‐step Likert scale, where 0 corresponds to *never or almost never* (less than 1 day), 1 corresponds to *sometimes or infrequently* (1–2 days), 2 corresponds to *occasionally or for a moderate amount of time* (3–4 days), and 4 corresponds to *most of the time or all of the time* (5–7 days), how they have been feeling in the past weeks. For the purpose of this study, the items of the original English version were translated into Italian by an expert and then independently back‐translated into English by a second translator. Discrepancies were discussed and resolved to ensure conceptual and linguistic equivalence. This scale was administered at T0 (*α* = .74 and *⍵* = .75), T1 (*α* = .76 and *⍵* = .77), and T2 (*α* = .71 and *⍵* = .72).

#### Personal savoring experiences

The participants of the savoring group were encouraged to describe their personal savoring experience in order to collect qualitative data immediately after each savoring exercise.

#### Evaluation of user–platform interaction

At the end of the intervention (T1), an ad hoc five‐item questionnaire was administered to assess participants' experience of interacting with the website, drawing on the Unified Theory of Acceptance and Use of Technology (UTAUT) model (Philippi et al., 2021). Responses were rated on a 7‐point Likert scale (1 = *not at all*; 7 = *very much*). The explorative factorial analysis enlightens two factors, one exploring the platform's ease of use (*α* = .86 and *⍵* = .86) and one focusing on perceived pleasantness (*α* = .87 and *⍵* = .82).

#### Perceived usefulness and pleasantness of the exercises after the training and at the 1‐month follow‐up and practice frequency at the 1‐month follow‐up

Immediately after the training (T1) and 1 month after the conclusion of the training, during the follow‐up phase (T2), participants were administered two ad hoc items aimed at investigating which exercise they found most useful and most pleasant on a seven‐step Likert scale where 1 corresponds to *not at all* and 7 to *very much*. The items were as follows: “Which exercise did you find most useful?” and “Which exercise did you find most pleasant?” Moreover, at T2 participants were administered one ad hoc item aimed at investigating how frequently they had freely practiced the various activities proposed during the intervention in the previous month (“In the past month, how often have you practiced the following exercises, on a scale from 1 to 7, where 1 corresponds to *not at all* and 7 to *very much*?”).

#### Perceived usefulness of the training

To assess the perceived usefulness of the overall training, an ad hoc item on a 7‐step Likert scale was administered (“Considering the entire training, how useful do you think it was for you on a scale from 1 to 7 (where 1 = *not at all* and 7 = *very much*)?”).

### Savoring and PE group activities

The entire intervention was designed and developed exclusively online. An ad hoc website was therefore designed and created via the WordPress platform (at the domain https://www.savor-ageing.it), accessible from any device (smartphone, tablet, or computer) with an Android, iOS, Windows, or macOS operating system. The goal was to create a website with simple graphics that was intuitive and usable. Participants accessed the site with a username and password, and once participants entered the website, they could access the online questionnaires (through a link to Qualtrics) and the activity for the savoring group (Table 1) and for the PE group (Table 2).

**TABLE 2:** Description of the PE group's activities.

Week	Themes	Purpose	Brief description
1	Listening to music	Eliciting positive emotional induction by listening to music.	Participants are invited to choose and listen to a music track from a selection of three. The use of music to induce positive emotions has been previously investigated by other studies (Vieillard & Bigand, 2014).
1	Watching a video	Eliciting positive emotional induction by watching a video.	Participants are invited to watch a brief video from *Mamma Mia!* movie Previous studies have demonstrated that viewing video content with humorous elements can induce positive emotions (Rottenberg et al., 2007; Yeo et al., 2020).
2	Engaging in an activity	Engaging participants in a pleasant activity without seeking to elicit savoring or emotional reflection.	Participants are invited to practice a pleasant activity on their own. Previous studies have demonstrated that being involved in positive activity can increase well‐being (Lyubomirsky & Layous, 2013).
2	Gratitude narrative	Inducing gratitude through listening to a narrative, without proposing emotional reflection and awareness of physical sensations.	Participants listen to a narrative about gratitude. Previous studies have utilized listening to narratives to induce positive emotions (Savard et al., 2024).
3	Engaging in an activity with someone	Engaging participants in a pleasant activity without seeking to elicit savoring or emotional reflection.	Participants are invited to practice a pleasant activity with someone else. Previous studies have demonstrated that being involved in positive activity can increase well‐being (Lyubomirsky & Layous, 2013).
3	Hope narrative	Inducing hope through listening to a narrative without proposing emotional reflection and awareness of physical sensations.	Participants listen to a narrative about hope. Previous studies have utilized listening to narratives to induce positive emotions (Savard et al., 2024).

The savoring group completed six savoring activities, each following the temporal focus of savoring (past, present, and future; Bryant & Veroff, 2007), which are described in Table 1. In the meantime, the PE group completed six emotional induction activities, which are described in Table 2. All participants received an e‐mail reminder every time a new activity was published on the website (on Mondays and Thursdays). Each activity started with a brief breathing exercise that was proposed to help participants calm down and focus on each activity.

### Data analysis

Data analysis was conducted using Jamovi 2.6.44 and STATA 19, and the data were screened for outliers and missing data. Group differences regarding demographic information, satisfaction with life (SWLS), positive and negative affect (SPANE‐P and SPANE‐N), flourishing (FS), and depression (CES‐D) were examined at baseline using the Student *t* test for independent samples (for continuous variables) and chi‐square tests (for categorical variables). To examine longitudinal changes across the three assessment points (T0–T2), linear mixed‐effects models (LMMs) were estimated for each outcome variable. Time, group condition (savoring vs. PE group), and their interaction were entered as fixed effects, whereas participants were included as a random intercept to account for repeated observations nested within individuals. Models were estimated using restricted maximum likelihood (REML). LMMs were selected because they appropriately account for the non‐independence of repeated observations and allow inclusion of all available longitudinal data under the missing at random assumption. Estimated marginal means were calculated to facilitate interpretation of longitudinal trajectories. Full model estimates and additional exploratory within‐group comparisons are reported in Appendix S1.

Furthermore, descriptive analyses were conducted to assess user experience in terms of both the perceived usefulness and pleasantness of the exercises at T1 and T2 and the perceived usefulness and pleasantness of the whole training.

Thematic analysis was employed to examine the qualitative data (Braun & Clarke, 2006). In order to address the research question, this method identifies, interprets, and organizes patterns of meaning (themes) across the dataset. It enabled the efficient synthesis of a large volume of textual material through a deductive approach, while still allowing unexpected themes to emerge inductively from the data. Relevant excerpts were systematically identified and then grouped into potential themes, which were subsequently refined, defined, and labeled by two independent reviewers. Finally, the resulting themes were further explored through narrative analysis.

## RESULTS

The characteristics of the final sample are presented in Table 3.

**TABLE 3:** Participants' baseline characteristics in the final sample.

		Savoring group (*N* = 36)	PE group (*N* = 40)	Statistic	*p*	Effect size
Age, *M* (*SD*)		70.20 (5.19)	69.60 (4.50)	*t*(74) = 0.48	.627	*d* = .112
Gender, *N* (%)	Male	9 (25%)	10 (25%)	*χ* ^2^(1) = 0.00	1.00	*φ* = 0.00
	Female	27 (75%)	30 (75%)			
Education level, *N* (%)	Middle school	3 (8%)	1 (3%)	*χ* ^2^(4) = 15.4	.004^a^	*V* = .450
	Senior high school	29 (80%)	20 (50%)			
	Bachelor's degree	–	3 (7%)			
	Master's degree	3 (8%)	16 (40%)			
	PhD	1 (4%)	–			
Marital status, *N* (%)	Single	1 (4%)	2 (5%)	*χ* ^2^(4) = 2.73	.604	*V* = .189
	Cohabitant	4 (11%)	2 (5%)			
	Married	18 (50%)	26 (65%)			
	Divorced	6 (16%)	4 (10%)			
	Widowed	7 (19%)	6 (15%)			
Employment status, *N* (%)	Worker	2 (5%)	1 (3%)	*χ* ^2^(3) = 4.27	.234	*V* = .237
	Retired	33 (91%)	36 (90%)			
	Unemployed	1 (4%)	–			
	Other	–	3 (7%)			
Baseline scores, *M* (*SD*)	SWLS (range: 7–35)	25.39 (5.83)	25.25 (5.75)	*t*(74) = 0.10	.917	*d* = .024
	SPANE‐P (range: 6–30)	21.58 (3.97)	21.27 (3.48)	*t*(74) = 0.36	.719	*d* = .082
	SPANE‐N (range: 6–30)	12.67 (3.85)	12.60 (3.61)	*t*(74) = 0.07	.938	*d* = .017
	FS (range: 8–56)	44.67 (5.41)	45.27 (6.96)	*t*(74) = −0.42	.674	*d* = −.096
	CES‐D (range: 0–30)	7.67 (4.13)	7.42 (4.77)	*t*(74) = 0.23	.815	*d* = .054

^a^ Significant (*p* < .05).

Abbreviations: CES‐D, Center for Epidemiologic Studies‐Depression Scale; FS, Flourishing Scale; M, mean; SD, standard deviation; SPANE‐N, Scale of Positive and Negative Experiences–Negative affect; SPANE‐P, Scale of Positive and Negative Experiences–Positive affect; SWLS, Satisfaction With Life Scale.

Data screening indicated that there were no missing values or outliers in the sample. Independent‐samples *t* tests (for continuous variables) and chi‐square tests (for categorical variables) were conducted to examine potential baseline differences between the two groups in the demographic information. The only significant difference was observed for education level, with a higher proportion of participants in the PE group holding a Master's degree compared to the savoring group. To examine baseline differences between groups in the psychological dimensions, independent‐samples *t* tests were conducted. No significant group differences were observed across any of the variables (Table 3).

### Effectiveness of the savoring intervention

**FIGURE 4:**
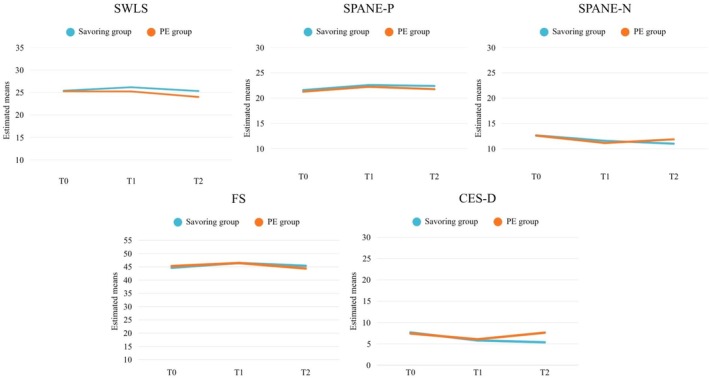
Scores of satisfaction with life (SWLS), positive affect (SPANE‐P), negative affect (SPANE‐N), flourishing (FS), and depression (CES‐D) at T0 (pre‐intervention), T1 (post‐intervention), and T2 (1‐month follow‐up) in the savoring and PE groups.

Linear mixed‐effects models were conducted to examine longitudinal changes in subjective well‐being, psychological well‐being, and depressive symptoms across the three assessment points (T0–T2). Time, group condition, and their interaction were entered as fixed effects, whereas participant was included as a random intercept. Descriptive statistics and linear mixed‐effects model results are reported in Table 4, whereas Figure 4 displays the estimated trajectories across assessment points. No significant time × group interactions emerged for satisfaction with life (SWLS), positive affect (SPANE‐P), negative affect (SPANE‐N), or flourishing (FS). Significant overall effects of time were observed for negative affect (SPANE‐N) and depressive symptoms. For depressive symptoms (CES‐D), the time × group interaction approached significance, suggesting differential longitudinal trajectories between groups. Estimated marginal means (Figure 4) suggested a progressive reduction in depressive symptoms in the savoring condition, whereas reductions in the PE group were not maintained at follow‐up. Detailed data are available in Appendix S1 (Tables S1 and S2).

**TABLE 4:** Descriptive statistics and linear mixed‐effects model results across assessment points.

	Savoring group *M* (*SD*)			PE group *M* (*SD*)			Time effect		Time × Group	
	T0	T1	T2	T0	T1	T2	*p*	*χ* ^2^	*p*	*χ* ^2^
SWLS	25.39	26.17	25.31	25.25	25.23	24.00	.641	0.89	.629	0.93
	(5.83)	(5.21)	(5.70)	(5.75)	(5.52)	(6.29)				
SPANE‐P	21.58	22.58	22.40	21.28	22.23	21.77	.283	2.53	.870	0.28
	(3.97)	(3.55)	(4.31)	(3.48)	(3.58)	(3.87)				
SPANE‐N	12.67	11.58	11.03	12.60	11.15	11.66	.027^a^	7.23	.324	2.25
	(3.85)	(3.74)	(3.43)	(3.61)	(3.39)	(3.50)				
FS	44.67	46.44	45.31	45.28	46.45	44.77	.086	4.92	.367	2.00
	(5.41)	(5.08)	(5.23)	(6.96)	(4.71)	(5.13)				
CES‐D	7.67	5.81	5.40	7.43	6.07	7.23	.007^a^	10.08	.053	5.87
	(4.13)	(4.49)	(3.53)	(4.77)	(3.80)	(4.40)				

^a^ Significant (*p* < .05).

Abbreviations: CES‐D, Center for Epidemiologic Studies‐Depression Scale; FS, Flourishing Scale; M, mean; SD, standard deviation; SPANE‐N, Scale of Positive and Negative Experiences–Negative affect; SPANE‐P, Scale of Positive and Negative Experiences–Positive affect; SWLS, Satisfaction With Life Scale.

To facilitate the interpretation of temporal patterns, exploratory within‐group comparisons were examined. Results are reported in Tables 5 and 6. Exploratory within‐group analyses suggested more consistent improvements over time in the savoring condition. Specifically, significant reductions emerged for negative affect (SPANE‐N) and depressive symptoms (CES‐D), particularly at follow‐up (T2). In addition, flourishing (FS) significantly increased from baseline to post‐intervention (T1) only in the savoring group. In the PE condition, changes were generally smaller and non‐significant at follow‐up, although a short‐term reduction in negative affect was observed at T1. Overall, these findings suggest that the savoring intervention may have promoted more sustained improvements in emotional well‐being over time compared to the PE condition.

**TABLE 5:** Estimated within‐group T0‐T1 changes.

	Savoring group			PE group		
	T1 vs. T0 (b)	*p*	*z*	T1 vs. T0 (b)	*p*	*z*
SWLS	0.78	.420	.81	−0.03	.978	−0.03
SPANE‐P	1.00	.132	1.51	0.95	.132	1.51
SPANE‐N	−1.08	.080	−1.75	−1.45	.014^a^	−2.47
FS	1.78	.027^a^	2.21	1.18	.124	1.54
CES‐D	−1.86	.015^a^	−2.44	−1.35	.063	−1.86

^a^ Significant (*p* < .05).

*Note*: Values represent marginal effects estimated from linear mixed models and indicate changes from baseline (T0) within each intervention group.

Abbreviations: CES‐D, Center for Epidemiologic Studies‐Depression Scale; FS, Flourishing Scale; SPANE‐N, Scale of Positive and Negative Experiences–Negative affect; SPANE‐P, Scale of Positive and Negative Experiences–Positive affect; SWLS, Satisfaction With Life Scale.

**TABLE 6:** Estimated within‐group T0‐T2 changes.

	Savoring group			PE group		
	T2 vs. T0 (b)	*p*	*z*	T2 vs. T0 (b)	*p*	*z*
SWLS	−0.02	.981	−0.02	−1.32	.168	−1.38
SPANE‐P	0.80	.234	1.19	0.34	.605	0.52
SPANE‐N	−1.65	.008^a^	−2.64	−0.73	.233	−1.19
FS	0.72	.374	0.89	−0.88	.271	−1.10
CES‐D	−2.30	.003^a^	−2.98	0.20	.791	0.26

^a^ Significant effect (*p* < .05).

*Note*: Values represent marginal effects estimated from linear mixed models and indicate changes from baseline (T0) within each group.

Abbreviations: CES‐D, Center for Epidemiologic Studies‐Depression Scale; FS, Flourishing Scale; M, mean; SD, standard deviation; SPANE‐N, Scale of Positive and Negative Experiences–Negative affect; SPANE‐P, Scale of Positive and Negative Experiences–Positive affect; SWLS, Satisfaction With Life Scale.

### Qualitative analysis of the personal savoring experiences

With regard to the thematic analyses, Table S3 summarizes the main themes that emerged from the participants' comments collected at the end of each savoring activity. The most recurrent thematic areas were intense positive emotions, affective and relational connection, physical and bodily sensations, relaxation and well‐being, and contact with nature. In particular, concerning the theme of intense positive emotions, participants reported experiences of general positive feelings such as joy, serenity, satisfaction, and gratitude. With regard to relational connections, participants reported experiences involving meaningful moments with loved ones (e.g., partner, sons and daughters, grandchildren, and friends), anticipated acts of kindness they intended to perform in the future, and expressions of gratitude toward those who support them daily and who had undertaken significant actions on their behalf. The exercises that most strongly elicited this theme were positive reminiscence (Activity 1), the gratitude letter (Activity 4), and the act of kindness (Activity 6) (see Table S3 for further details). The theme of bodily sensations emerged in participants' references to physiological changes, such as an accelerated heartbeat or slowed breathing following the activity. Others described concrete physical sensations, including the wind on their face, the burning of tears in their eyes, and the warmth of their hands. The theme of relaxation and well‐being encompasses all comments in which participants explicitly stated that they experienced a sense of calm and well‐being after completing the activity, thereby emphasizing its beneficial effects. Finally, the theme of contact with nature refers to all comments in which participants recalled, engaged in, or imagined activities carried out in natural settings, appreciating their beauty and specific details. In Appendix S1, there are also some examples of the comments posted after each activity by the savoring group (Table S4).

Table S5 (see Appendix S1) reports the frequencies with which participants in the savoring group indicated experiencing specific emotions in the comments provided at the end of each activity. As shown in Table S5, the reported emotions were predominantly positive. The frequencies reported in Table S5 refer to the number of times participants mentioned that emotion. Overall, the most frequently reported positive emotions among participants who completed the savoring intervention were joy (*N* = 87), followed by serenity (*N* = 71) and happiness (*N* = 55). An exception was observed in the gratitude letter exercise (Activity 4), where a limited number of participants reported experiencing feelings of sadness (*N* = 6) and nostalgia (*N* = 8).

### Evaluation of user‐platform interaction

Regarding the evaluation of user–platform interaction, both the savoring and PE groups reported high levels of ease of use of the website (range: 1–7 for each subscale; savoring group: *M* = 6.15; *SD* = 0.93; PE group: *M* = 6.36; *SD* = 0.76). Similarly, both groups reported high levels of perceived pleasantness (savoring group: *M* = 5.65; *SD* = 0.94; PE group: *M* = 5.65; *SD* = 1.0), suggesting that participants reported that interacting with the website was intuitive and enjoyable.

### Perceived usefulness and pleasantness of the exercises after the training and at the 1‐month follow‐up and practice frequency at the 1‐month follow‐up

Distinct preferences emerged between the two conditions regarding the perceived usefulness and pleasantness of the exercises. At T1, the savoring group primarily identified the gratitude letter (*N* = 12) and positive reminiscence (*N* = 8) as the most useful activities, while rating positive reminiscence as the most pleasant (*N* = 12). Conversely, the PE group endorsed gratitude (*N* = 5) and hope narratives (*N* = 5) as the most useful, whereas listening to music (*N* = 14) and watching a video (*N* = 11) were perceived as the most pleasant. At the 1‐month follow‐up (T2), preferences shifted slightly. The savoring group consistently favored positive reminiscence (*N* = 8) and getting absorbed during an activity (*N* = 7) across both usefulness and pleasantness dimensions. In the PE group, the gratitude narrative prominently emerged as both the most useful (*N* = 22) and pleasant (*N* = 13) exercise, alongside engaging in pleasant activities (*N* = 7). Furthermore, both groups reported autonomous practice of the interventions during the follow‐up period. The savoring group most frequently continued the “getting absorbed during an activity” exercise, whereas the PE group predominantly engaged with the “listening to/reading the hope narrative” task.

### Perceived usefulness of the training

At T1, participants' ratings of the overall usefulness of the training were generally high in both groups. In the savoring group, some participants rated it as *extremely useful* (*N* = 5), and the most frequent ratings were *very useful* (*N* = 16) and *useful* (*N* = 11), whereas only a few rated the intervention as *not at all useful* (*N* = 2) or *slightly useful* (*N* = 1). For example, C.U. reported, “I found the course useful, very interesting, and easy to put into practice whenever you want, as it is not too long. It also has a positive effect on your mood and helps you recognize the positive things that happen to you. Greater overall awareness.” And E.B. stated, “It was a relaxing and pleasant experience that helped me strengthen my ability to be present and aware in the here and now. Thank you for the opportunity!” In the PE group, a few participants rated it as *extremely useful* (*N* = 2), and the majority of the participants rated it as *very useful* (*N* = 18) and *useful* (*N* = 12), with very few participants giving the lowest ratings. These results indicate that participants in both groups perceived the activities as generally beneficial. Moreover, participants' explicit opinions and suggestions about the intervention were also analyzed qualitatively at both T1 and T2 (see Table S6). Overall, the activities of both groups were highly appreciated. Both sets of activities appeared to encourage reflection and greater self‐awareness, although such effects were more frequently reported in the savoring group. Notably, participants in the PE group tended to provide cognitive evaluations of the activities' quality rather than describing tangible personal benefits, suggesting a deeper level of emotional engagement and transformative experience among those in the savoring group. Finally, several participants mentioned possible future applications of both sets of activities, highlighting their perceived usefulness and overall positive impact.

## DISCUSSION

As aging is often accompanied by a decline in functional and physical abilities and a reduction in subjective and psychological well‐being, strategies that foster resilience become increasingly important (Friedman et al., 2017). In this regard, a savoring intervention may play a key role in promoting healthy and positive aging, as the ability to savor and enhance positive emotions and experiences can help counterbalance these age‐related challenges (Smith & Bryant, 2019). For example, recent evidence suggests that savoring‐based interventions can be particularly effective in promoting emotional well‐being and mitigating negative affect in older adults, including those facing grief or social isolation (Basic & Bryant, 2025). Therefore, online savoring interventions may be particularly important and well suited for older adults who are socially isolated or have limited mobility (Smith et al., 2020), as they may benefit less from the positive effects of physical activity on well‐being. This aspect is also supported by the work of Grossi et al. (2020), which highlights the relevance of positive technology for older adults—particularly those who are socially isolated and may lack access to alternative forms of support. In this framework, the objective of the present study is to investigate the effectiveness of a savoring intervention in promoting subjective and psychological well‐being and decreasing depression in a sample of older adults, compared with positive emotion enhancement training for a PE group, in order to test the efficacy of the specific effects. Moreover, this RCT seeks to understand the perception and user experience of the specific web‐based training. The findings are very encouraging and partially support our hypothesis.

### Subjective well‐being

Regarding the primary outcomes, such as the effectiveness of the intervention on increasing subjective well‐being (Hypothesis 1), no significant improvement was observed both in the cognitive component of life satisfaction and in the affective component. The lack of significant change in life satisfaction was probably due to the short duration of the training (Villani et al., 2023). Although the training's brevity (six activities over 3 weeks) supported high adherence, it was insufficient to bring about lasting changes in participants' overall cognitive evaluation of their lives. Evidence from longer interventions suggests that more time is needed to achieve enduring effects (Salces‐Cubero et al., 2019). This result can also be better understood considering the reflections offered by Wilson et al. (2025), who argue that in the older adult population, present‐focused savoring may be particularly effective in influencing the cognitive dimension of subjective well‐being. Therefore, future studies should consider incorporating a greater number of present‐focused exercises to maximize potential benefits on life satisfaction. As far as the affective component of subjective well‐being is concerned, positive affect did not significantly improve in either the savoring or the PE group, and no sustained effects emerged at follow‐up. Likewise, no significant differences were observed between groups over time. The interpretation of this result should also take into account that participants already exhibited relatively high baseline levels of positive affect, possibly linked to their engagement in cultural programs such as the Universities of the Third Age. Furthermore, the mere decision to participate in a well‐being intervention may have triggered positive emotions connected to the act of self‐care.

Moreover, as far as changes in negative affect are concerned, both groups showed a significant decrease in negative emotions over time. In particular, participants in the PE group showed a significant reduction in negative affect immediately after the intervention, whereas participants in the savoring group showed a significant decrease between baseline and the 1‐month follow‐up. However, no significant between‐group differences emerged in the reduction of negative emotions, in line with previous online interventions conducted by Villani et al. (2023) and Yu et al. (2020). The finding observed in the PE group is also consistent with previous positive emotion induction interventions, showing that training focused on inducing positive emotions may lead to short‐term reductions in negative affect (Sin & Lyubomirsky, 2009). At the same time, these findings suggest that savoring may contribute more specifically to longer term reductions in negative affect by encouraging older adults to shift their attention from unpleasant or distressing thoughts toward positive experiences and emotions. Through the intentional appreciation of meaningful moments, savoring may help reframe daily experiences, promoting a more balanced emotional perspective and reducing the intensity or frequency of negative feelings (Barbara Fredrickson, 1998; Bryant & Veroff, 2007). In this sense, savoring training may foster more sustained effects than the mere induction of positive emotions. Savoring activities, by enhancing awareness and amplification of positive experiences and their associated bodily sensations, may facilitate a deeper processing of positive states and, in turn, a more stable reduction in negative emotions. In contrast, the PE activities primarily focused on eliciting positive emotions without providing guidance on how to attend to, sustain, or cognitively elaborate these experiences, which may explain their more short‐term effects. Notably, the PE group did not show significant changes from baseline to the 1‐month follow‐up in either life satisfaction or the affective components of subjective well‐being, suggesting that participants experienced only temporary decreases in negative affect that were insufficient to produce lasting improvements in subjective well‐being over the follow‐up period.

### Psychological well‐being

Concerning the effects of the savoring intervention on participants' psychological well‐being (Hypothesis 2), within‐subjects analyses showed that flourishing significantly increased in the short term only in the savoring group, whereas no significant change was observed in the PE group. This finding is consistent with prior work demonstrating that savoring‐based exercises can produce meaningful short‐term gains in well‐being (Bryant & Veroff, 2007; Jose et al., 2012). However, this effect was not maintained at the one‐month follow‐up, and no significant differences emerged between groups over time, despite the presence of a significant time effect, contrary to what had been expected based on the literature describing the potential effects of manipulating savoring levels on psychological well‐being (Smith & Hollinger‐Smith, 2015; Friedman et al., 2017). This finding may once again be correlated to the decision to participate in a well‐being intervention for themselves that may have elicited a deep connection to personal value and psychological well‐being. The structure and delivery of the activities in the PE condition were similar to those of the savoring group (including audio and text tracks with instructions and ongoing contact via e‐mail with the researchers). It is therefore plausible that these factors fostered a sense of actively contributing to a well‐being‐oriented intervention, which may have influenced participants' perceptions and outcomes. However, no significant changes were observed in the PE group from baseline to the post‐intervention and the 1‐month follow‐up, suggesting that the mere induction of positive emotions was not sufficient to produce short and lasting improvements in psychological well‐being. In this regard, previous research indicates that present‐focused savoring, in particular, produces significant effects on older adults' psychological well‐being (Wilson et al., 2025). Accordingly, a possible explanation for the lack of long‐term significant results in the savoring group may lie in insufficient attention to this orientation. At the same time, the literature also emphasizes the importance of reminiscence for well‐being in later life (Sherman & Peak, 1991). One of the present study's strengths is the attention given to all three temporal orientations of savoring, rather than focusing on only one. Future research could therefore move in the direction of maintaining attention to all three temporal dimensions of savoring while also expanding the emphasis placed on the present and past orientation. Another important element to consider is the concept of person‐intervention fit, according to which the effectiveness of an intervention also depends on the degree of alignment between individual characteristics and its features (Lyubomirsky et al., 2005; Lyubomirsky & Layous, 2013; Sheldon & Lyubomirsky, 2007). The current study's sample comprised individuals who were already active and socially engaged, reporting elevated baseline levels of psychological well‐being. It is therefore plausible that the absence of a significant increase in well‐being may be attributable to these initially high levels. This result is in line with the findings of Smith and Hollinger‐Smith (2015), who concluded that the relationship between savoring and psychological well‐being is stronger among individuals with lower resilience. This interpretation is further supported by Sutipan et al. (2017), who stress that positive psychology interventions are not equally effective for all older adults but tend to yield greater benefits for those with more room for improvement in well‐being. Finally, it is worth noting that the literature currently available on the effects of savoring in older adults, especially in online interventions, remains limited and is characterized by a small number of controlled clinical trials (Stephens et al., 2025).

### Depression

Concerning the effectiveness of the intervention in decreasing depression, the third hypothesis was partially supported (Hypothesis 3). From a between‐subjects perspective, a marginally significant interaction effect was found in the reduction of depression levels over the three measurement points, favoring the savoring group. These findings suggest that a self‐help savoring intervention can effectively reduce depressive symptoms even 1 month after completion in a sample of active and healthy older adults. Although both groups showed changes over the three assessment moments, only the savoring group continued to show a significant decrease in depression levels both immediately after the intervention and at the one‐month follow‐up. These findings are supported by the existing literature on the topic. Smith and Hanni (2019) demonstrated that an online savoring intervention conducted with an older adult population can reduce negative thoughts and behaviors that may interfere with one's ability to savor and can diminish positive emotions. Specifically, they observed a decrease in depressive symptoms among participants who maintained high intervention fidelity over time, across multiple assessment points, including the 1‐ and 3‐month follow‐ups. These results align with the present study's findings, as depression levels decreased following the intervention and remained stable at follow‐up assessments. Several studies have further supported this finding by demonstrating an association between savoring and greater adaptive capacity in critical situations. Specifically, among older adults, higher savoring ability has been found to predict fewer depressive symptoms (Bryant, 2003; Ramsey & Gentzler, 2014; Smith & Hollinger‐Smith, 2015). Moreover, daily time‐savoring has been shown to moderate the associations between daily solitude and depressive mood, daily solitude and loneliness, and daily solitude and somatic symptoms (Wallimann et al., 2024). In contrast, the PE group showed no significant reduction in depressive symptoms across the three measurement points, with a slight increase in depression levels observed at the 1‐month follow‐up, suggesting a lack of sustained effects over time for this condition. Although the PE intervention was designed to elicit positive emotions, it did not include mechanisms for regulating or prolonging these emotional experiences, as in the savoring intervention. This difference may help explain the more enduring improvements observed in the savoring group, highlighting its potential unique role in producing longer‐lasting benefits for mood and emotional well‐being.

Overall, the present quantitative findings are partially consistent with those reported by Pancini et al. (2023), which also examined a 3‐week online savoring intervention in older adults. Methodologically, the studies differ substantially: Pancini et al. (2023) used a within‐subjects pilot design with a small sample and focused mainly on proximal outcomes (e.g., savoring strategies and positive emotions), whereas the present study employed a larger randomized controlled design, included an active control condition and a one‐month follow‐up assessment, and assessed a broader range of outcomes, including subjective and psychological well‐being and depression. In line with Pancini et al. (2023), no significant improvements emerged in broader and more stable dimensions of well‐being, such as life satisfaction, possibly due to the short duration of the intervention. Moreover, whereas Pancini et al. (2023) did not find significant reductions in maladaptive outcomes (i.e., negative emotions), the present RCT revealed a significant between‐group effect in depressive symptoms, with a greater reduction in the savoring group over time and a decrease in negative emotions only in the savoring group. In this regard, this study extends prior research by providing stronger causal evidence, disentangling savoring from simple positive emotion induction, and highlighting its potential in reducing depressive symptoms over time.

The present quantitative findings are also consistent with those reported in online savoring interventions conducted with other age groups, particularly young adults. For example, Jiao et al. (2021) conducted an online study to examine the effects of a savoring exercise centered on positive emotions derived from “communication moments.” Their findings revealed a significant difference in favor of the experimental group, characterized by an increase in positive affect and a reduction in negative aspects. These results were further corroborated by Villani et al. (2023), who evaluated an online savoring intervention of similar duration to ours (six sessions over 3 weeks). Their study reported a significant time × group interaction effect post‐intervention, indicating a substantial increase in positive affect for the experimental group. Furthermore, Yu et al. (2020) implemented an online savoring intervention via Facebook involving a sample of university students. The results demonstrated the intervention's effectiveness in significantly reducing depressive symptoms within the experimental group, although these gains were not maintained at follow‐up.

### Perceived impact of the intervention and platform and qualitative results

As participants of both groups accessed the online training through a website specifically designed to be simple and intuitive for an older population, the user–platform interaction has also been assessed. Both groups positively rated the user‐platform interaction with the website, finding it easy and pleasant to use. At the conclusion of the training (T1), participants in the savoring group reported that the most useful exercise was the gratitude letter (Activity 4), whereas the most enjoyable was the positive reminiscence task (Activity 1). The gratitude letter was considered particularly valuable due to the strong emotional engagement it elicited and the opportunity it provided to express deep feelings toward loved ones. Writing down their emotions in this letter evoked in participants a profound sense of gratitude and fulfillment, fostering positive emotions and strengthening the connection between personal experience and emotional well‐being (Boggiss et al., 2020; Killen & Macaskill, 2015). The positive reminiscence exercise was rated as the most enjoyable, as it offered the opportunity to relive happy and meaningful memories from the past, as previously shown by Villani et al. (2023). Regarding the perceived usefulness of the intervention, participants in both groups considered the training beneficial and described it as an opportunity for reflection. However, the savoring group perceived it as more useful and reported greater benefits, including enhanced awareness of positive emotions and increased calm and relaxation. In contrast, the PE group mainly described the training as reinforcing existing habits, with limited evidence of deeper change or integration into daily emotional regulation. In this regard, the qualitative analyses suggest that this savoring intervention may represent a particularly promising approach for older adults. Compared to the emotion‐induction activities (PE intervention), these exercises seem to enhance engagement with positive experiences by increasing awareness of emotional processes and the embodied aspect of the experience. These aspects may represent key elements that make savoring abilities particularly useful for emotion regulation and for the prioritization of meaningful goals in older adults, as suggested by previous research (Carstensen & Charles, 1998; Charles & Carstensen, 2009; Pancini et al., 2023; Urry & Gross, 2010).

### Strengths and limitations of the study

This study is situated within a promising yet still underexplored field of research and has several strengths. First of all, from a methodological perspective, the present RCT was prospectively registered on ClinicalTrials.gov and achieved an adequate sample size, additionally including an active PE group to rigorously test the effectiveness of the online savoring intervention in an older population. The use of a PE group allowed for a more accurate assessment of the intervention's efficacy, isolating the specific impact of savoring activities from non‐specific factors related to mere engagement in an online positive emotion enhancement intervention. Another strength of the study lies in the integrated collection of both quantitative and qualitative data, which enabled a deeper understanding of participants' experiences with the platform, their reflections on the training process, and their feedback on the proposed activities. This mixed‐methods approach provided a more nuanced picture of participants' engagement, highlighting which exercises were most appreciated and which continued to be practiced in the months following the intervention's completion. Furthermore, the presence of engaging and intuitive exercises contributed to maintaining a remarkably low dropout rate, an especially noteworthy result considering the well‐documented reluctance of older adults to engage with technological tools (Failli et al., 2023). This outcome is even more significant given that the 3‐week savoring intervention was designed to be completed entirely independently, without direct facilitator support. These findings suggest that the intervention successfully engaged and motivated older adults to complete the training. A key strength of the present study lies in its theoretical contribution to the positive psychology and aging literature, as it provides empirical support for the specific role of savoring, showing that actively engaging with and savoring positive experiences across time can reduce depressive symptoms and foster longer term benefits, particularly in later life, compared to the mere experience of positive emotions without their intentional amplification (Bryant et al., 2021; Chen et al., 2026; Fredrickson, 2004). From a clinical and practical perspective, this study highlights the potential of self‐help online savoring interventions as scalable tools to promote mental health in aging populations. Notably, the intervention proved effective in reducing depressive symptoms over time, even in a sample of active and psychologically healthy older adults. This suggests that savoring may function as a preventive resource also for individuals with subclinical symptoms or lower baseline well‐being. This finding contributes to supporting the theoretical framework of the two‐continua model (Westerhof & Keyes, 2010), which posits that mental illness and mental health lie on two parallel continua and that it is possible to intervene on either dimension to achieve overall benefits. This evidence therefore supports the indication to implement targeted well‐being strategies, such as savoring, in order to achieve benefits also in terms of reducing psychological distress (Sin & Lyubomirsky, 2009). Moreover, the online format enhances accessibility, making the intervention particularly suitable for older adults facing social isolation, reduced mobility, or limited access to traditional psychological services. In this sense, the findings support the integration of savoring‐based approaches within digital mental health and positive aging frameworks. Future RCTs should consider combining online savoring activities with group‐based experiences to foster social connection and implementing such interventions in structured contexts such as hospitals and retirement homes. Moreover, the observed longer term reduction in depressive symptoms highlights the potential value of incorporating savoring into broader mental health promotion and positive aging programs.

From a methodological perspective, a potential limitation lies in the absence of a no‐intervention control group. Future studies could include, alongside an active control group, useful for controlling for potential effects due to time or extra‐experimental conditions, a passive control group in order to more effectively account for effects related to participation in a highly engaging intervention and the perceived monitoring by the researchers, aspects that could influence the interpretation of this intervention's effects. Among the limitations, the recruited sample exhibited high levels of well‐being and low levels of depression. Future research could investigate whether a savoring intervention is more effective than a positive emotional induction program in clinical elderly populations, who typically present higher levels of depression and lower levels of well‐being. This is particularly relevant from both a theoretical and clinical perspective. Indeed, the literature shows that positive psychology interventions tend to produce stronger effects among individuals with lower initial levels of well‐being or among those with depressive symptoms (Ruini, 2021; Sin & Lyubomirsky, 2009). A further limitation concerns the sample, which was recruited only from Universities of the Third Age. Future studies could test the intervention in more diverse populations. Lastly, the study focused on an Italian sample from a restricted geographical area. Future research could expand the study to include participants from across the country.

## CONCLUSION

In conclusion, the present RCT provides robust evidence that a 3‐week online savoring intervention, *Savor‐Aging*, can effectively reduce depressive symptoms and negative emotions in healthy older adults over time. By encouraging individuals to intentionally focus on and prolong positive experiences, savoring can enhance emotional awareness, self‐reflection, and appreciation of meaningful life moments. These findings highlight the critical role of savoring as a protective psychological mechanism capable of counteracting the possible emotional decline associated with aging. Importantly, they suggest that savoring interventions can foster lasting psychological resources, support adaptive emotion regulation, and promote mental health in older adulthood. Overall, this study reinforces the practical value of integrating structured savoring activities within digital mental health and positive aging programs, offering an accessible, engaging, and evidence‐based approach to promoting emotional well‐being and preventing depressive symptoms in later life.

## CONFLICT OF INTEREST STATEMENT

The authors declare no conflicts of interest.

## ETHICS STATEMENT

This study has been conducted in compliance with the Declaration of Helsinki and approved by the Ethical Committee for Research in Psychology of the Department of Psychology of the Università Cattolica del Sacro Cuore of Milan (Protocol Number: 128/24). All participants signed an informed consent form before beginning the research about the research protocol, data protection, and privacy according to the General Data Protection Regulation (GDPR; EU 2016/679). The study's objectives, confidentiality, and anonymity were described, and volunteers were given full authority to complete the study. All methods were performed in accordance with the relevant guidelines and regulations in the Declaration.

## Supporting information


**Table S1.** Full fixed‐effect estimates from the linear mixed‐effects models for intervention outcomes across assessment points.
**Table S2.** Estimated marginal means across assessment points by both groups.
**Table S3.** Qualitative analysis of the savoring exercises (number of participants).
**Table S4.** Participants' quotes after the savoring exercises (savoring group).
**Table S5.** Most frequent emotions experienced by the savoring group during the savoring exercises (number of participants).
**Table S6.** Qualitative analysis of final reflections in the savoring group with participants' quotes.

**Data S1.** Supplementary Information.

## Data Availability

The dataset generated and analyzed during this study is available from the corresponding author upon reasonable request.

## References

[aphw70184-bib-0001] Andresen, E. M. , Malmgren, J. A. , Carter, W. B. , & Patrick, D. L. (1994). Screening for depression in well older adults: Evaluation of a short form of the CES‐D (Center for Epidemiologic Studies Depression Scale). American Journal of Preventive Medicine, 10(2), 77–84. 10.1016/S0749-3797(18)30622-6 8037935

[aphw70184-bib-0002] Baird, B. M. , Lucas, R. E. , & Donnellan, M. B. (2010). Life satisfaction across the lifespan: Findings from two nationally representative panel studies. Social Indicators Research, 99(2), 183–203. 10.1007/s11205-010-9584-9 21113322 PMC2990956

[aphw70184-bib-0003] Basic, A. , & Bryant, F. B. (2025). Harnessing relational savoring to mitigate grief: Evaluating the effectiveness of an intervention for bereaved older adults. The Journal of Positive Psychology, 1‐13, 1–13. 10.1080/17439760.2025.2582001

[aphw70184-bib-0004] Blanchflower, D. G. , & Oswald, A. J. (2008). Is well‐being U‐shaped over the life cycle? Social Science & Medicine, 66(8), 1733–1749. 10.1016/j.socscimed.2008.01.030 18316146

[aphw70184-bib-0005] Boggiss, A. L. , Consedine, N. S. , Brenton‐Peters, J. M. , Hofman, P. L. , & Serlachius, A. S. (2020). A systematic review of gratitude interventions: Effects on physical health and health behaviors. Journal of Psychosomatic Research, 135, 110165. 10.1016/j.jpsychores.2020.110165 32590219

[aphw70184-bib-0006] Bouwman, T. , van Tilburg, T. , & Aartsen, M. (2019). Attrition in an online loneliness intervention for adults aged 50 years and older: Survival analysis. JMIR Aging, 2(2), e13638. 10.2196/13638 31518268 PMC6715013

[aphw70184-bib-0007] Braun, V. , & Clarke, V. (2006). Using thematic analysis in psychology. Qualitative Research in Psychology, 3(2), 77–101. 10.1191/1478088706qp063oa

[aphw70184-bib-0008] Bryant, F. B. (2003). Savoring Beliefs Inventory (SBI): A scale for measuring beliefs about savouring. Journal of Mental Health, 12(2), 175–196. 10.1080/0963823031000103489

[aphw70184-bib-0009] Bryant, F. B. , Chadwick, E. D. , & Kluwe, K. (2011). Understanding the processes that regulate positive emotional experience: Unsolved problems and future directions for theory and research on savoring. International Journal of Wellbeing, 1(1), 107–126. 10.5502/ijw.v1i1.18

[aphw70184-bib-0010] Bryant, F. B. , Osowski, K. A. , & Smith, J. L. (2021). Gratitude as a mediator of the effects of savoring on positive adjustment to aging. The International Journal of Aging and Human Development, 92(3), 275–300. 10.1177/0091415020919999 32370635

[aphw70184-bib-0011] Bryant, F. B. , & Veroff, J. (2007). Savoring: A new model of positive experience. Erlbaum Associates.

[aphw70184-bib-0012] Carstensen, L. L. , & Charles, S. T. (1998). Emotion in the second half of life. Current Directions in Psychological Science, 7(4), 144–149. 10.1111/1467-8721.ep10774734

[aphw70184-bib-0013] Charles, S. T. , & Carstensen, L. L. (2009). Social and emotional aging. Annual Review of Psychology, 61, 383–409. 10.1146/annurev.psych.60.110707.163659 PMC3950961 19575618

[aphw70184-bib-0014] Chen, P. H. , Tung, H. H. , Wu, Y. C. , & Sie, J. (2026). Effectiveness of savoring interventions: A systematic review and meta‐analysis of randomized controlled trials. Applied Psychology. Health and Well‐Being, 18(2), e70134. 10.1111/aphw.70134 41797412 PMC12968602

[aphw70184-bib-0015] Cohn, M. A. , Pietrucha, M. E. , Saslow, L. R. , Hult, J. R. , & Moskowitz, J. T. (2014). An online positive affect skills intervention reduces depression in adults with type 2 diabetes. The Journal of Positive Psychology, 9(6), 523–534. 10.1080/17439760.2014.920410 25214877 PMC4157680

[aphw70184-bib-0016] Colombo, D. , Pavani, J. B. , Quoidbach, J. , Baños, R. M. , Folgado‐Alufre, M. , & Botella, C. (2024). Savouring the present to better recall the past. Journal of Happiness Studies, 25(1), 20. 10.1007/s10902-024-00718-4

[aphw70184-bib-0017] Corno, G. , Molinari, G. , & Baños, R. M. (2016). Assessing positive and negative experiences: Validation of a new measure of well‐being in an Italian population. Rivista di Psichiatria, 51(3), 110–115. 10.1708/2386.25595 27362822

[aphw70184-bib-0018] di Fabio, A. (2016). Flourishing Scale: Primo contributo alla validazione della versione italiana [Flourishing Scale: First contribution to the validation of the Italian version]. Counseling: Giornale Italiano di Ricerca e Applicazioni, 9(1).

[aphw70184-bib-0019] di Fabio, A. , & Busoni, L. (2009). Proprietà psicometriche della versione italiana della Satisfaction With Life Scale (SWLS) con studenti universitari. Counseling: Giornale Italiano di Ricerca e Applicazioni, 2(2), 201–211.

[aphw70184-bib-0021] Diener, E. D. , Emmons, R. A. , Larsen, R. J. , & Griffin, S. (1985). The satisfaction with life scale. Journal of Personality Assessment, 49(1), 71–75. 10.1207/s15327752jpa4901_13 16367493

[aphw70184-bib-0020] Diener, E. , Wirtz, D. , Tov, W. , Kim‐Prieto, C. , Choi, D. W. , Oishi, S. , & Biswas‐Diener, R. (2010). New well‐being measures: Short scales to assess flourishing and positive and negative feelings. Social Indicators Research, 97(2), 143–156. 10.1007/s11205-009-9493-y

[aphw70184-bib-0022] Failli, D. , Arpino, B. , & Marino, M. F. (2023). A finite mixture approach for the analysis of digital skills in Finland, Italy and Bulgaria: The role of socio‐economic factors. *arXiv preprint*. 10.48550/arXiv.2311.03801

[aphw70184-bib-0023] Folstein, M. F. , Folstein, S. E. , & McHugh, P. R. (1975). “Mini‐Mental State”: A practical method for grading the mental state of patients for the clinician. Journal of Psychiatric Research, 12(3), 189–198. 10.1016/0022-3956(75)90026-6 1202204

[aphw70184-bib-0024] Fredrickson, B. L. (1998). What good are positive emotions? Review of General Psychology, 2(3), 300–319. 10.1037/1089-2680.2.3.300 21850154 PMC3156001

[aphw70184-bib-0025] Fredrickson, B. L. (2004). The broaden–and–build theory of positive emotions. Philosophical Transactions of the Royal Society of London. Series B: Biological Sciences, 359(1449), 1367–1377. 10.1098/rstb.2004.1512 15347528 PMC1693418

[aphw70184-bib-0026] Fredrickson, B. L. , Arizmendi, C. , & van Cappellen, P. (2021). Same‐day, cross‐day, and upward spiral relations between positive affect and positive health behaviours. Psychology & Health, 36(4), 444–460. 10.1080/08870446.2020.1778696 32538212 PMC9536170

[aphw70184-bib-0027] Friedman, E. M. , Ruini, C. , Foy, R. , Jaros, L. , Sampson, H. , & Ryff, C. D. (2017). Lighten UP! A community‐based group intervention to promote psychological well‐being in older adults. Aging & Mental Health, 21(2), 199–205. 10.1080/13607863.2015.1093593 26460594 PMC5636191

[aphw70184-bib-0028] Gloria, C. T. , & Steinhardt, M. A. (2016). Relationships among positive emotions, coping, resilience and mental health. Stress and Health, 32(2), 145–156. 10.1002/smi.2589 24962138

[aphw70184-bib-0029] Grossi, G. , Lanzarotti, R. , Napoletano, P. , Noceti, N. , & Odone, F. (2020). Positive technology for elderly well‐being: A review. Pattern Recognition Letters, 137, 61–70. 10.1016/j.patrec.2019.02.017

[aphw70184-bib-0030] Heintzelman, S. J. , Kushlev, K. , Lutes, L. D. , Wirtz, D. , Kanippayoor, J. M. , Leitner, D. , Oishi, S. , & Diener, E. (2020). ENHANCE: Evidence for the efficacy of a comprehensive intervention program to promote subjective well‐being. Journal of Experimental Psychology: Applied, 26(2), 360–383. 10.1037/xap0000254 31657590

[aphw70184-bib-0031] Ho, H. C. Y. , Yeung, D. Y. , & Kwok, S. Y. C. L. (2014). Development and evaluation of the positive psychology intervention for older adults. The Journal of Positive Psychology, 9(3), 187–197. 10.1080/17439760.2014.888577

[aphw70184-bib-0033] Jiao, J. , Kim, S. , & Pitts, M. J. (2021). Promoting subjective well‐being through communication savoring. Communication Quarterly, 69(2), 152–171. 10.1080/01463373.2021.1901758

[aphw70184-bib-0034] Jose, P. E. , Lim, B. T. , & Bryant, F. B. (2012). Does savoring increase happiness? A daily diary study. The Journal of Positive Psychology, 7(3), 176–187. 10.1080/17439760.2012.671345

[aphw70184-bib-0035] Kang, H. (2021). Sample size determination and power analysis using the G*Power software. Journal of Educational Evaluation for Health Professions, 18, 17. 10.3352/jeehp.2021.18.17 34325496 PMC8441096

[aphw70184-bib-0036] Killen, A. , & Macaskill, A. (2015). Using a gratitude intervention to enhance well‐being in older adults. Journal of Happiness Studies, 16(4), 947–964. 10.1007/s10902-014-9542-3

[aphw70184-bib-0037] Lyubomirsky, S. , King, L. , & Diener, E. (2005). The benefits of frequent positive affect: Does happiness lead to success? Psychological Bulletin, 131(6), 803–855. 10.1037/0033-2909.131.6.803 16351326

[aphw70184-bib-0038] Lyubomirsky, S. , & Layous, K. (2013). How do simple positive activities increase well‐being? Current Directions in Psychological Science, 22(1), 57–62. 10.1177/0963721412471860

[aphw70184-bib-0039] Magni, E. , Binetti, G. , Bianchetti, A. , Rozzini, R. , & Trabucchi, M. (1996). Mini‐Mental State Examination: A normative study in Italian elderly population. European Journal of Neurology, 3(3), 198–202. 10.1111/j.1468-1331.1996.tb00423.x 21284770

[aphw70184-bib-0040] Measso, G. , Cavarzeran, F. , Zappalà, G. , Lebowitz, B. D. , Crook, T. H. , Pirozzolo, F. J. , Amaducci, L. A. , Massari, D. , & Grigoletto, F. (1993). The mini‐mental state examination: Normative study of an Italian random sample. Developmental Neuropsychology, 9(2), 77–85. 10.1080/87565649109540545

[aphw70184-bib-0041] Metitieri, T. , Geroldi, C. , Pezzini, A. , Frisoni, G. B. , Bianchetti, A. , & Trabucchi, M. (2001). The Itel‐MMSE: An Italian telephone version of the mini‐mental state examination. International Journal of Geriatric Psychiatry, 16(2), 166–167. 10.1002/1099-1166(200102)16:2<166::AID-GPS290>3.0.CO;2-M 11241721

[aphw70184-bib-0042] Pancini, E. , Pesce, F. , Scuzzarella, L. , & Villani, D. (2023). Promoting positive emotions in older adults: A self‐help relational savoring e‐intervention. In In International Conference on Human‐Computer Interaction (pp. 88–101). Springer Nature Switzerland. 10.1007/978-3-031-34866-2_7

[aphw70184-bib-0043] Philippi, P. , Baumeister, H. , Apolinário‐Hagen, J. , Ebert, D. D. , Hennemann, S. , Kott, L. , Lin, J. , Messner, E. M. , & Terhorst, Y. (2021). Acceptance towards digital health interventions–model validation and further development of the unified theory of acceptance and use of technology. Internet Interventions, 26, 100459. 10.1016/j.invent.2021.100459 34603973 PMC8463857

[aphw70184-bib-0044] Quoidbach, J. , Berry, E. V. , Hansenne, M. , & Mikolajczak, M. (2010). Positive emotion regulation and well‐being: Comparing the impact of eight savoring and dampening strategies. Personality and Individual Differences, 49(5), 368–373. 10.1016/j.paid.2010.03.041

[aphw70184-bib-0045] Radloff, L. S. (1977). The CES‐D scale: A self‐report depression scale for research in the general population. Applied Psychological Measurement, 1(3), 385–401. 10.1177/014662167700100306

[aphw70184-bib-0046] Ramírez, E. , Ortega, A. R. , Chamorro, A. , & Colmenero, J. M. (2014). A program of positive intervention in the elderly: Memories, gratitude and forgiveness. Aging & Mental Health, 18(4), 463–470. 10.1080/13607863.2013.848834 24229346

[aphw70184-bib-0047] Ramsey, M. A. , & Gentzler, A. L. (2014). Age differences in subjective well‐being across adulthood: The roles of savoring and future time perspective. The International Journal of Aging and Human Development, 78(1), 3–22. 10.2190/AG.78.1.b 24669507

[aphw70184-bib-0048] Rottenberg, J. , Ray, R. R. , & Gross, J. J. (2007 (in press)). Emotion elicitation using films. In J. A. Coan & J. J. B. Allen (Eds.), The handbook of emotion elicitation and assessment. Oxford University Press.

[aphw70184-bib-0049] Ruini, C. (2021). Positive psychology and clinical psychology: An integrated perspective. Springer.

[aphw70184-bib-0050] Ryff, C. D. (1989). Happiness is everything, or is it? Explorations on the meaning of psychological well‐being. Journal of Personality and Social Psychology, 57(6), 1069–1081. 10.1037/0022-3514.57.6.1069

[aphw70184-bib-0051] Salces‐Cubero, I. M. , Ramírez‐Fernández, E. , & Ortega‐Martínez, A. R. (2019). Strengths in older adults: Differential effect of savoring, gratitude and optimism on well‐being. Aging & Mental Health, 23(8), 1017–1024. 10.1080/13607863.2018.1471585 29781723

[aphw70184-bib-0052] Salces‐Cubero, I. M. , Ramírez‐Fernández, E. , & Ortega‐Martínez, A. R. (2025). The differential effect of training in humor, forgiveness, savoring, and meaning and purpose on the well‐being of older adults. Journal of Happiness Studies, 26(7), 122. 10.1007/s10902-025-00938-9

[aphw70184-bib-0053] Salovey, P. , Rothman, A. J. , Detweiler, J. B. , & Steward, W. T. (2000). Emotional states and physical health. American Psychologist, 55(1), 110–121. 10.1037/0003-066X.55.1.110 11392855

[aphw70184-bib-0054] Savard, M. A. , Merlo, R. , Samithamby, A. , Paas, A. , & Coffey, E. B. (2024). Approaches to studying emotion using physiological responses to spoken narratives: A scoping review. Psychophysiology, 61(11), e14642. 10.1111/psyp.14642 38961524

[aphw70184-bib-0055] Sheldon, K. M. , & Lyubomirsky, S. (2007). Is it possible to become happier? (And if so, how?). Social and Personality Psychology Compass, 1(1), 129–145. 10.1111/j.1751-9004.2007.00002.x

[aphw70184-bib-0056] Sherman, E. , & Peak, T. (1991). Patterns of reminiscence and the assessment of late life adjustment. Journal of Gerontological Social Work, 16(1–2), 59–74. 10.1300/J083v16n01_05

[aphw70184-bib-0057] Sin, N. L. , & Lyubomirsky, S. (2009). Enhancing well‐being and alleviating depressive symptoms with positive psychology interventions: A practice‐friendly meta‐analysis. Journal of Clinical Psychology, 65(5), 467–487. 10.1002/jclp.20593 19301241

[aphw70184-bib-0058] Smith, J. L. , Bihary, J. G. , O'Connor, D. , Basic, A. , & O'Brien, C. J. (2020). Impact of savoring ability on the relationship between older adults' activity engagement and well‐being. Journal of Applied Gerontology, 39(3), 323–331. 10.1177/0733464818765278 31478422

[aphw70184-bib-0059] Smith, J. L. , & Bryant, F. B. (2016). The benefits of savoring life: Savoring as a moderator of the relationship between health and life satisfaction in older adults. The International Journal of Aging and Human Development, 84(1), 3–23. 10.1177/0020731415598502 27655957

[aphw70184-bib-0060] Smith, J. L. , & Bryant, F. B. (2019). Enhancing positive perceptions of aging by savoring life lessons. Aging & Mental Health, 23(6), 762–770. 10.1080/13607863.2018.1450840 29553804

[aphw70184-bib-0061] Smith, J. L. , & Hanni, A. A. (2019). Effects of a savoring intervention on resilience and well‐being of older adults. Journal of Applied Gerontology, 38(1), 137–152. 10.1177/0733464817711425 28380722

[aphw70184-bib-0062] Smith, J. L. , Harrison, P. R. , Kurtz, J. L. , & Bryant, F. B. (2014). Nurturing the capacity to savor: Interventions to enhance the enjoyment of positive experiences. In The Wiley Blackwell handbook of positive psychological interventions (pp. 42–65). Wiley Blackwell. 10.1002/9781118315927.ch3

[aphw70184-bib-0063] Smith, J. L. , & Hollinger‐Smith, L. (2015). Savoring, resilience, and psychological well‐being in older adults. Aging & Mental Health, 19(3), 192–200. 10.1080/13607863.2014.920296 25471325

[aphw70184-bib-0064] Sözeri‐Varma, G. (2012). Depression in the elderly: Clinical features and risk factors. Aging and Disease, 3(6), 465–471. 10.14336/AD.2012.0300465 23251852 PMC3522513

[aphw70184-bib-0065] Stephens, J. E. , Mertz, L. , & Smith, J. L. (2025). Within‐and between‐person effects of savoring ability and well‐being in older adults: A longitudinal study. Journal of Happiness Studies, 26(1), 3. 10.1007/s10902-024-00845-5

[aphw70184-bib-0066] Stockwell, S. , Stubbs, B. , Jackson, S. E. , Fisher, A. , Yang, L. , & Smith, L. (2021). Internet use, social isolation and loneliness in older adults. Ageing and Society, 41(12), 2723–2746. 10.1017/S0144686X20000550

[aphw70184-bib-0067] Stone, A. A. , Broderick, J. E. , Wang, D. , & Schneider, S. (2020). Age patterns in subjective well‐being are partially accounted for by psychological and social factors associated with aging. PLoS ONE, 15(12), e0242664. 10.1371/journal.pone.0242664 33264331 PMC7710094

[aphw70184-bib-0068] Sutipan, P. , Intarakamhang, U. , & Macaskill, A. (2017). The impact of positive psychological interventions on well‐being in healthy elderly people. Journal of Happiness Studies, 18(1), 269–291. 10.1007/s10902-015-9711-z

[aphw70184-bib-0069] Tibubos, A. N. , Reinwarth, A. C. , Reiner, I. , Werner, A. M. , Wild, P. S. , Münzel, T. , König, J. , Lackner, K. J. , Pfeiffer, N. , & Beutel, M. E. (2025). Psychological indicators for healthy aging: Validation of the German short version of Ryff's scales of psychological well‐being (SPWB). Journal of Patient‐Reported Outcomes, 9(1), 25. 10.1186/s41687-025-00854-9 39998720 PMC11861829

[aphw70184-bib-0070] Urry, H. L. , & Gross, J. J. (2010). Emotion regulation in older age. Current Directions in Psychological Science, 19(6), 352–357. 10.1177/0963721410388395

[aphw70184-bib-0071] van Cappellen, P. , & Fredrickson, B. L. (2025). Authentic embodied positive emotions: An experiential introduction to the broaden‐and‐build theory. In More activities for teaching positive psychology: A guide for instructors (pp. 13–23). American Psychological Association. 10.1037/0000417-002

[aphw70184-bib-0072] Vieillard, S. , & Bigand, E. (2014). Distinct effects of positive and negative music on older adults' auditory target identification performances. Quarterly Journal of Experimental Psychology, 67(11), 2225–2238. 10.1080/17470218.2014.910167 24871301

[aphw70184-bib-0073] Villani, D. , Pancini, E. , Pesce, F. , & Scuzzarella, L. (2023). Savoring life during pandemic: An online intervention to promote well‐being in emerging adults. BMC Psychology, 11(1), 196. 10.1186/s40359-023-01225-z 37403128 PMC10318654

[aphw70184-bib-0074] Wallimann, M. , Peleg, S. , & Pauly, T. (2024). Time‐savoring moderates associations of solitude with depressive mood, loneliness, and somatic symptoms in older adults' daily life. Applied Psychology. Health and Well‐Being, 16(3), 1497–1515. 10.1111/aphw.12538 38520051

[aphw70184-bib-0075] Westerhof, G. J. , & Keyes, C. L. (2010). Mental illness and mental health: The two continua model across the lifespan. Journal of Adult Development, 17(2), 110–119. 10.1007/s10804-009-9082-y 20502508 PMC2866965

[aphw70184-bib-0076] Wilson, J. M. , Strough, J. , & Shook, N. J. (2025). Understanding age differences in well‐being: Pathways from present time orientation to mindfulness and savoring the moment. The International Journal of Aging and Human Development. 10.1177/00914150241313358 39819008

[aphw70184-bib-0077] World Health Organization . (2023). Mental health of older adults. https://www.who.int/news-room/fact-sheets/detail/mental-health-of-older-adults

[aphw70184-bib-0078] World Health Organization . (2025). Mental health of older adults. https://www.who.int/news-room/fact-sheets/detail/mental-health-of-older-adults

[aphw70184-bib-0079] Yeo, N. L. , White, M. P. , Alcock, I. , Garside, R. , Dean, S. G. , Smalley, A. J. , & Gatersleben, B. (2020). What is the best way of delivering virtual nature for improving mood? An experimental comparison of high definition TV, 360 video, and computer generated virtual reality. Journal of Environmental Psychology, 72, 101500. 10.1016/j.jenvp.2020.101500 33390641 PMC7772948

[aphw70184-bib-0080] Yu, S. C. , Sheldon, K. M. , Lan, W. P. , & Chen, J. H. (2020). Using social network sites to boost savoring: Positive effects on positive emotions. International Journal of Environmental Research and Public Health, 17(17), 6407. 10.3390/ijerph17176407 32887521 PMC7503456

