# Supplementary material for: Savor‐Aging: The Art of Savoring Positive Emotions in Older Adulthood—A Randomized Controlled Trial
Source: Appl Psychol Health Well Being. 2026 Jul 4;18(4):e70184. doi: 10.1111/aphw.70184 (PMC13332415; doi:10.1111/aphw.70184)
Supplement: Supplementary file 1 — Table S1. Full fixed‐effect estimates from the linear mixed‐effects models for intervention outcomes across assessment points. Table S2. Estimated marginal means across assessment points by both groups. Table S3. Qualitative analysis of the savoring exercises (number of participants). Table S4. Participants' quotes after the savoring exercises (savoring group). Table S5. Most frequent emotions experienced by the savoring group during the savoring exercises (number of participants). Table S6. Qualitative analysis of final reflections in the savoring group with participants' quotes. [file APHW-18-0-s001.docx]

**APPENDIX 1**

**Table S1.** Full fixed-effect estimates from the linear mixed-effects models for intervention outcomes across assessment points.

| **Outcome** | **Predictor** | **b** | **SE** | **z** | **p** | **95% CI** | |
| --- | --- | --- | --- | --- | --- | --- | --- |
| *SWLS* | Time T1 | 0.778 | 0.964 | 0.810 | 0.420 | -1.111 | 2.667 |
|  | Time T2 | -0.024 | 0.973 | -0.020 | 0.981 | -1.930 | 1.883 |
|  | Group (PE) | -0.139 | 1.312 | -0.110 | 0.916 | -2.711 | 2.433 |
|  | T1 × PE | -0.803 | 1.328 | -0.600 | 0.546 | -3.406 | 1.801 |
|  | T2 × PE | -1.295 | 1.364 | -0.950 | 0.342 | -3.970 | 1.379 |
|  | _cons | 25.39 | 0.95 | 26.67 | 0.00 | 23.52 | 27.25 |
|  |  |  |  |  |  |  |  |
|  | N observations | 222 |  |  |  |  |  |
|  | N participants | 76 |  |  |  |  |  |
|  | Random intercept variance | 15.916 | 3.619 |  |  | 10.193 | 24.852 |
|  | Residual variance | 16.717 | 1.981 |  |  | 13.252 | 21.087 |
|  |  |  |  |  |  |  |  |
|  | ICC (95% CI) | 0.488 (0.357 - 0.620) |  |  |  |  |  |
|  |  |  |  |  |  |  |  |
| *SPANE-P* | Time T1 | 1.000 | 0.664 | 1.510 | 0.132 | -0.302 | 2.302 |
|  | Time T2 | 0.797 | 0.670 | 1.190 | 0.234 | -0.516 | 2.111 |
|  | Group (PE) | -0.308 | 0.873 | -0.350 | 0.724 | -2.020 | 1.404 |
|  | T1 × PE | -0.050 | 0.916 | -0.050 | 0.956 | -1.844 | 1.744 |
|  | T2 × PE | -0.456 | 0.940 | -0.490 | 0.627 | -2.299 | 1.386 |
|  | _cons | 21.583 | 0.634 | 34.060 | 0.000 | 20.341 | 22.825 |
|  |  |  |  |  |  |  |  |
|  | N observations | 222 |  |  |  |  |  |
|  | N participants | 76 |  |  |  |  |  |
|  | Random intercept variance | 6.514 | 1.566 |  |  | 4.066 | 10.435 |
|  | Residual variance | 7.941 | 0.945 |  |  | 6.289 | 10.027 |
|  |  |  |  |  |  |  |  |
|  | ICC (95% CI) | 0.451 (0.317 - 0.591) |  |  |  |  |  |
|  |  |  |  |  |  |  |  |
| *SPANE-N* | Time T1 | -1.083 | 0.619 | -1.750 | 0.080 | -2.296 | 0.130 |
|  | Time T2 | -1.649 | 0.625 | -2.640 | 0.008 | -2.874 | -0.425 |
|  | Group (PE) | -0.067 | 0.824 | -0.080 | 0.936 | -1.681 | 1.548 |
|  | T1 × PE | -0.367 | 0.853 | -0.430 | 0.667 | -2.039 | 1.305 |
|  | T2 × PE | 0.917 | 0.876 | 1.050 | 0.295 | -0.800 | 2.634 |
|  | _cons | 12.667 | 0.598 | 21.190 | 0.000 | 11.495 | 13.838 |
|  |  |  |  |  |  |  |  |
|  | N observations | 222 |  |  |  |  |  |
|  | N participants | 76 |  |  |  |  |  |
|  | Random intercept variance | 5.965 | 1.398 |  |  | 3.768 | 9.444 |
|  | Residual variance | 6.896 | 0.818 |  |  | 5.466 | 8.700 |
|  |  |  |  |  |  |  |  |
|  | ICC (95% CI) | 0.464 (0.332 - 0.601) |  |  |  |  |  |
|  |  |  |  |  |  |  |  |
| *FS* | Time T1 | 1.778 | 0.806 | 2.210 | 0.027 | 0.198 | 3.358 |
|  | Time T2 | 0.723 | 0.814 | 0.890 | 0.374 | -0.872 | 2.319 |
|  | Group (PE) | 0.608 | 1.279 | 0.480 | 0.634 | -1.898 | 3.114 |
|  | T1 × PE | -0.603 | 1.111 | -0.540 | 0.587 | -2.781 | 1.575 |
|  | T2 × PE | -1.606 | 1.143 | -1.410 | 0.160 | -3.845 | 0.633 |
|  | _cons | 44.667 | 0.928 | 48.160 | 0.000 | 42.849 | 46.485 |
|  |  |  |  |  |  |  |  |
|  | N observations | 222 |  |  |  |  |  |
|  | N participants | 76 |  |  |  |  |  |
|  | Random intercept variance | 19.274 | 3.918 |  |  | 12.940 | 28.708 |
|  | Residual variance | 11.697 | 1.397 |  |  | 9.256 | 14.782 |
|  |  |  |  |  |  |  |  |
|  | ICC (95% CI) | 0.622 (0.502 - 0.729) |  |  |  |  |  |
|  |  |  |  |  |  |  |  |
| *CES-D* | Time T1 | -1.861 | 0.764 | -2.440 | 0.015 | -3.359 | -0.363 |
|  | Time T2 | -2.296 | 0.771 | -2.980 | 0.003 | -3.807 | -0.784 |
|  | Group (PE) | -0.242 | 0.973 | -0.250 | 0.804 | -2.148 | 1.665 |
|  | T1 × PE | 0.511 | 1.053 | 0.490 | 0.628 | -1.553 | 2.576 |
|  | T2 × PE | 2.496 | 1.081 | 2.310 | 0.021 | 0.377 | 4.615 |
|  | _cons | 7.667 | 0.706 | 10.860 | 0.000 | 6.283 | 9.050 |
|  |  |  |  |  |  |  |  |
|  | N observations | 222 |  |  |  |  |  |
|  | N participants | 76 |  |  |  |  |  |
|  | Random intercept variance | 7.417 | 1.902 |  |  | 4.487 | 12.262 |
|  | Residual variance | 10.512 | 1.257 |  |  | 8.315 | 13.289 |
|  |  |  |  |  |  |  |  |
|  | ICC (95% CI) | 0.413 (0.278 - 0.564) |  |  |  |  |  |
|  |  |  |  |  |  |  |  |

*Note*. Estimates are derived from linear mixed-effects models with participant included as a random intercept. T0 and the savoring condition were used as reference categories. ICC = intraclass correlation coefficient.

SWLS: Satisfaction With Life Scale; SPANE-P: Scale of Positive and Negative Experiences – Positive affect; SPANE-N: Scale of Positive and Negative Experiences – Negative affect; FS: Flourishing Scale; CES-D: Center for Epidemiologic Studies-Depression Scale.

**Table S2.** Estimated marginal means across assessment points by both groups.

| **Outcome** | **Time** | **Savoring group** | | | |  | **PE group** | | | |
| --- | --- | --- | --- | --- | --- | --- | --- | --- | --- | --- |
|  |  | **Mean** | **SE** | **95% CI** | |  | **Mean** | **SE** | **95% CI** | |
| *SWLS* | T0 | 25.39 | 0.95 | 23.52 | 27.25 |  | 25.25 | 0.90 | 23.48 | 27.02 |
|  | T1 | 26.17 | 0.95 | 24.30 | 28.03 |  | 25.23 | 0.90 | 23.45 | 27.00 |
|  | T2 | 25.37 | 0.96 | 23.48 | 27.25 |  | 23.93 | 0.95 | 22.08 | 25.79 |
| *SPANE-P* | T0 | 21.58 | 0.63 | 20.34 | 22.83 |  | 21.58 | 0.63 | 20.34 | 22.83 |
|  | T1 | 22.58 | 0.63 | 21.34 | 23.83 |  | 22.58 | 0.63 | 21.34 | 23.83 |
|  | T2 | 22.38 | 0.64 | 21.13 | 23.64 |  | 22.38 | 0.64 | 21.13 | 23.64 |
| *SPANE-N* | T0 | 12.67 | 0.60 | 11.50 | 13.84 |  | 12.60 | 0.57 | 11.49 | 13.71 |
|  | T1 | 11.58 | 0.60 | 10.41 | 12.75 |  | 11.15 | 0.57 | 10.04 | 12.26 |
|  | T2 | 11.02 | 0.60 | 9.83 | 12.20 |  | 11.87 | 0.59 | 10.70 | 13.03 |
| *FS* | T0 | 44.67 | 0.93 | 42.85 | 46.48 |  | 45.28 | 0.88 | 43.55 | 47.00 |
|  | T1 | 46.44 | 0.93 | 44.63 | 48.26 |  | 46.45 | 0.88 | 44.73 | 48.17 |
|  | T2 | 45.39 | 0.93 | 43.56 | 47.22 |  | 44.39 | 0.91 | 42.60 | 46.18 |
| *CES-D* | T0 | 7.67 | 0.71 | 6.28 | 9.05 |  | 7.43 | 0.67 | 6.11 | 8.74 |
|  | T1 | 5.81 | 0.71 | 4.42 | 7.19 |  | 6.08 | 0.67 | 4.76 | 7.39 |
|  | T2 | 5.37 | 0.71 | 3.97 | 6.77 |  | 7.63 | 0.70 | 6.24 | 9.01 |

*Note*. Values represent estimated marginal means derived from the linear mixed-effects models.

SWLS: Satisfaction With Life Scale; SPANE-P: Scale of Positive and Negative Experiences – Positive affect; SPANE-N: Scale of Positive and Negative Experiences – Negative affect; FS: Flourishing Scale; CES-D: Center for Epidemiologic Studies-Depression Scale.

**Table S3.** Qualitative analysis of the savoring exercises (number of participants).

| **Theme** |  | **Positive reminiscence** | **Life Lessons** | **Absorption during an activity** | **Gratitude letter** | **Anticipation of positive events** | **Making a kind gesture** |
| --- | --- | --- | --- | --- | --- | --- | --- |
| *Intense positive emotions* |  | 39 | 15 | 32 | 43 | 38 | 25 |
| *Affective and relational connection* |  | 29 | 13 | 8 | 20 | 18 | 35 |
| *Bodily and physical sensations* |  | 3 | 4 | 3 | 6 | 12 | 4 |
| *Relaxation and well-being* |  | 0 | 0 | 36 | 5 | 6 | 18 |
| *Contact with nature* |  | 6 | 2 | 17 | 2 | 10 | 0 |

**Table S4.** Participants' quotes after the savoring exercises (savoring group).

| **Activity** | **Quotes** |
| --- | --- |
| **1**  **Positive reminiscence** | *“I was on holiday at the seaside with my daughter, already an adult, and I remember how grateful I was to be able to take this holiday, how proud I am of my daughter, how lucky I am. Thinking about this holiday, I felt immense joy and gratitude. My heartbeat quickened and I would love to repeat a trip like this.*” A.G.  “*As a memory, I chose the Christmas Eve I spent with my aunt and uncle when I was 8 or 10 years old. My aunt and uncle didn't have children, so I went to Mass with my aunt and then slept at their house in a room that was my bedroom. I visualized the room with the furniture and a photograph of myself hanging on the wall. I could smell the scent of freshly laundered sheets and feel the warmth of the house, especially the room, which smelled clean, and the warmth of my aunt and uncle. My breathing slowed down, my body relaxed, especially my legs*.” N.G. |
| **2**  **Life Lessons** | “*The experience I am referring to is when I discovered I was pregnant without being married, almost 50 years ago. Although I felt a little afraid, the emotion I felt at the time was the awareness that from then on I would no longer be alone. The feeling I have now is a very sweet emotion at the thought that my son and his brother who came after him are now good fathers and loving sons to me. Physically, my eyes are burning with the tears that are welling up*.” B.Z.  “*The event that changed my life was the birth of my daughter. I remember the moment she was born perfectly, and strangely, I don't remember any pain, only what I thought as soon as she was taken out of the delivery room and I was waiting to be taken to the ward. I was laughing to myself and saying, You've done something unique that no other human being can do the same. I felt like an artist who is aware that they have created the masterpiece of their life.*” P.V. |
| **3**  **Absorption during an activity** | “*Walking in the spring drizzle, but with a temperature of 13 degrees outside. Relaxation, the beauty of the blossoming trees, serenity, the feeling of rain on my face, life*.” C.C.  “*Reading a book... takes me away from everyday life, cheers me up even if the content isn't particularly entertaining, relaxes me, helps me sleep and gives me a feeling of well-being. Reading a book, not thinking and feeling transported to a faraway place.*” R.B. |
| **4**  **Gratitude letter** | “*I thought long and hard about who to dedicate the letter to, and in the end it was her, my mum, the person I can never thank enough. I feel above all great affection and gratitude towards her. She has always been discreetly by my side in happy and difficult times, giving me strength, independence, self-esteem and always lots of love. The strongest emotion I felt while writing the letter was like talking to my mum and remembering so many moments. I maintained this feeling of gratitude and esteem in the next exercise, feeling it particularly in my chest. My breathing was slow and deep. I would like to add that my mum is no longer with us, but it is as if she were always close to my whole family.*”. Z.K.  “*The letter I wrote is addressed to someone who is no longer with us, so I couldn't deliver it. I chose this person because of the lessons they taught me, their (spiritual) closeness, and the strength and courage they gave me. At first, it was difficult to start writing, but then it was like a river in flood. Beautiful emotions that warmed my heart. I felt relaxed, happy and full of strength (even physical strength). I will try to repeat the exercise by writing a letter and then delivering it to the person concerned. If I succeed, it would be almost a miracle, but I will let you know*.” C.U. |
| **5**  **Anticipation of positive events** | *“I thought about the trip I will start tomorrow morning in Tuscany, a region I particularly love. I enjoy the train journey, fast but slow, which allows me to admire the landscape, to think, to admire the beauty of our places. I think about the pleasant company of friends, the chats, but also the monuments and museums I will see, and I feel calm and serene*.” C.T.  “*Soon my garden will be bursting with color. Some flowers are already giving off a wonderful scent. I cultivate my little garden (with the help of an expert) with passion and energy. I spend every spare moment pruning, weeding and keeping the garden tidy. I feel at peace with the world and satisfied with the results. When I am in the garden, I am happy and relaxed, and seeing my plants bloom fills me with joy.*”. P.V. |
| **6**  **Making a kind gesture** | “*I thought I would surprise a dear friend who had her birthday a few days ago. In the next few days, her husband will undergo chemotherapy, so I will surprise her by inviting her for coffee and giving her a book, which for me is the best gift. Making others feel good and offering a few words of comfort is also good for us. I imagined the pleasure and excitement this friend will feel, and immediately I felt the same way myself. I will cheer her up, and I will benefit from it too.*” T.R.  “*Here were two gestures, one involving my wife and the other my grandson. The first was that I undertook to cook a dish of seitan and mushrooms that we would eat last Sunday when we returned from Milan after seeing a theatre show. The second was to be very helpful this morning when my grandson Jacopo woke up and changed his mind about the time I had to take him to school. In both cases, the people were very happy and peaceful, which was my goal, and I felt good about it.*” A. T. |

**Table S5.** Most frequent emotions experienced by the savoring group during the savoring exercises (number of participants).

| **Emotion** | **Positive reminiscence** | **Life lessons** | **Absorption during an activity** | **Gratitude letter** | **Anticipation of positive events** | **Making a kind gesture** | **Total** |
| --- | --- | --- | --- | --- | --- | --- | --- |
| *Joy* | 10 | 9 | 9 | 16 | 24 | 19 | 87 |
| *Serenity* | 9 | 6 | 6 | 17 | 19 | 17 | 71 |
| *Gratitude* | 7 | 10 | 6 | 18 | 0 | 11 | 52 |
| *Happiness* | 6 | 8 | 0 | 16 | 10 | 15 | 55 |
| *Relaxation* | 5 | 0 | 22 | 7 | 15 | 0 | 49 |
| *Pride* | 0 | 5 | 0 | 0 | 0 | 6 | 11 |
| *Nostalgia* | 0 | 0 | 0 | 8 | 0 | 0 | 8 |
| *Love* | 0 | 0 | 0 | 6 | 0 | 0 | 6 |
| *Sadness* | 0 | 0 | 0 | 6 | 0 | 0 | 6 |
| *Hope* | 0 | 0 | 0 | 0 | 8 | 0 | 8 |

**Table S6.** Qualitative analysis of final reflections in the savoring group with participants’ quotes.

| **Themes** | **Savoring group** | **Participants’ quotes** |
| --- | --- | --- |
| **Reflection and self-awareness** | **General reflection (7):** Participants reported that the experience allowed them to engage in general reflection.  **Reflection on their life (13):** Participants mentioned that the activity encouraged them to reflect on their own lives.  **Awareness of their life (1):** One participant noted that the experience helped them gain greater awareness of their own life.  **Awareness of the present moment (1):** One participant shared that the activity increased their awareness of the present moment. | *“It was good because sometimes we act and reflect without thinking deeply. These exercises helped me to reflect more on the actions I take.”* N.R.  *“In general, I think this was a moment of self-reflection, a chance to stop and look at how far we have come. It was certainly a pleasant experience and, above all, beneficial for our self-esteem.”* M.S. |
| **Strengths** | **Interesting (10):** Participants described the experience as interesting.  **Useful (10):** Participants reported that they found the experience useful.  **Engaging (1):** One participant found the activity engaging and stimulating. | *“I believe that all the proposed activities are useful in helping people lead a more pleasant and less stressful life.”* L.F.  *“I found the course useful, very interesting, and easy to put into practice whenever you want, as it is not too long. It also has a positive effect on your mood and helps you recognize the positive things that happen to you. Greater overall awareness.”* C.U. |
| **Perceived benefits** | **Pleasantness (12):** Participants reported experiencing a general sense of pleasantness during the activity.  **Relaxation (5):** Several participants stated that the activity made them feel relaxed.  **Learning (2):** A few participants indicated that they learned something new from the experience.  **Savoring and amplification (3):** Participants mentioned that the activity helped them savor the experience and amplify positive emotions.  **Valorization (of oneself, of what exists, and of one's own path) (4):** Participants described a sense of valorization, appreciating themselves, what surrounds them, and their personal journey. | *“It was pleasant and relaxing.”* G.F.  *“It has greatly assisted me in further savoring both past and future moments of happiness and joy.”* M.C. |
| **Future orientation** | **Desire for in-person sessions (2):** Participants expressed a desire to experience the activity through in-person sessions.  **Desire for group sharing and connection (2):** Participants indicated an interest in sharing the experience with a group to foster connection.  **Desire to repeat the exercises (1):** One participant stated they would like to repeat the exercises in the future.  **Desire to know the results (1):** One participant expressed curiosity about knowing the outcomes of the activity. | *“A very interesting journey, as far as I am concerned. I wonder if it would also be useful to be able to do the same journey in person and perhaps in a group..”* L.C.  *“I would like to receive the results of this process.”* M.C. |
| **Mixed comments** | **Perceived lack of usefulness (1):** One participant described the training and its activities as useless.  **Difficulty benefiting due to pet loss (1):** One participant reported that the recent loss of their pet made it difficult to fully benefit from the program.  **Reflection leading to dissatisfaction (1):** One participant stated that the training led her to reflect on her life and recognize feelings of unhappiness and dissatisfaction. | *“I found it useless.”* D.F.  *“I'm sorry, I just lost my cat, my best companion, and I can't think straight.”* E.V. |
