# Supplementary material for: Savor‐Aging: The Art of Savoring Positive Emotions in Older Adulthood—A Randomized Controlled Trial
Source: Appl Psychol Health Well Being. 2026 Jul 4;18(4):e70184. doi: 10.1111/aphw.70184 (PMC13332415; doi:10.1111/aphw.70184)
Supplement: Supplementary file 2 — Data S1. Supplementary Information. [file APHW-18-0-s002.docx]

**APPENDIX 2—Validity indicators of the questionnaires administered at T0, T1, T2**

*Satisfaction With Life Scale (SWLS)*

To examine the structural validity of the Satisfaction With Life Scale (SWLS) in the present sample, a confirmatory factor analysis (CFA) was conducted at T0 using a maximum likelihood estimator. The hypothesized one-factor model demonstrated adequate fit to the data: χ²(5) = 17.25, p = .004, CFI = .951, TLI = .902, RMSEA = .179 [90% CI: .092, .276]. Although the RMSEA value exceeded the conventional cutoff of .08, this index is known to be particularly sensitive to small sample sizes and models with few degrees of freedom, which may have inflated its value in the present study (Kenny et al., 2015). All standardized factor loadings were statistically significant (p < .001) and ranged from .696 to .872, indicating that each item contributed meaningfully and strongly to the latent construct of life satisfaction.

*Scale of Positive and Negative Experience (SPANE)*

A CFA was conducted at T0 to examine the structural validity of the Scale of Positive and Negative Experience (SPANE) in the present sample, using a maximum likelihood estimator. Consistently with the original two-factor structure proposed by Diener et al. (2010), a model with two correlated factors (positive emotions, SPANE-P, and negative emotions, SPANE-N) was tested. The model showed good fit: χ²(53) = 73.80, p = .031, CFI = .958, TLI = .948, RMSEA = .072 [90% CI: .023, .109]. The two factors were negatively correlated (r = -.731, p < .001), consistent with theoretical expectations. All factor loadings were statistically significant (p < .001) and ranged from .357 to .792.

*Flourishing Scale (FS)*

A CFA was conducted at T0 to examine the structural validity of the Flourishing Scale (FS) in the present sample, using a maximum likelihood estimator. The hypothesized one-factor model showed marginal fit: χ²(20) = 51.10, p < .001, CFI = .900, TLI = .860, RMSEA = .143 [90% CI: .095, .192]. Although some indices did not reach conventional thresholds, this pattern is consistent with findings from previous validation studies of the FS in older adult samples, which have similarly reported suboptimal RMSEA values and lower factor loadings for certain items (Didino et al., 2019). These results may partly reflect the sensitivity of fit indices to small sample sizes (Kenny et al., 2015). All factor loadings were statistically significant (p < .001) and ranged from .525 to .909.

*Center for Epidemiologic Studies-Depression Scale (CES-D)*

A CFA was conducted at T0 to examine the factorial structure of the CES-D in the present sample using a maximum likelihood estimator. The hypothesized one-factor model showed acceptable-to-marginal fit: χ²(35) = 54.90, p = .017, CFI = .823, TLI = .773, RMSEA = .086, [90% CI: .037, .129]. Although some fit indices did not reach conventional cutoffs, all factor loadings were statistically significant and ranged from .290 to .686, a range consistent with those reported in prior CFA-based validation studies of the CES-D-10 (Mohebbi et al., 2018; Padmanabhanunni & Pretorius, 2023). Notably, Mohebbi et al. (2018) reported standardized factor loadings between .28 and .64 in healthy older adults using the same one-factor model. Given the relatively small sample size and the well-documented sensitivity of global fit indices to sample characteristics, these findings should be interpreted cautiously. Importantly, the aim of the present study was not to validate the factorial structure of the CES-D, but rather to use a consistent measure of depressive symptoms across the three assessment moments (T0, T1, and T2) in order to examine longitudinal changes over time. Accordingly, the same scale structure was retained across all measurement occasions, in line with the recommended practice for repeated-measures designs (see also Kilburn et al., 2018). Furthermore, the CES-D demonstrated adequate internal consistency throughout the study: at T0 (α = .74, ω = .75), T1 (α = .76, ω = .77), and T2 (α = .71, ω = .72), supporting its reliability for repeated measurement in the present sample. These values are consistent with those reported in prior studies employing the CES-D-10 across diverse samples (Andresen et al., 1994; Kilburn et al., 2018; Padmanabhanunni & Pretorius, 2023) and fall within the broader reliability range documented in the CES-D literature (α = .70–.95; Niu et al., 2021). The concurrent use of both Cronbach's alpha and McDonald's omega provides a more comprehensive account of scale reliability, as omega is less sensitive to violations of the assumption of tau-equivalence that alpha requires (Flora, 2020).

*Evaluation of user–platform interaction*

Because this was an ad hoc five-item questionnaire developed for the present study to assess participants’ experience interacting with the website, an exploratory factor analysis (EFA) was conducted to examine its underlying structure. The questionnaire was conceptually informed by the Unified Theory of Acceptance and Use of Technology (UTAUT) model (Philippi et al., 2021).

The EFA was conducted using the minimum residual extraction method with Varimax rotation. Results suggested a two-factor solution. The first factor reflected perceived ease of use of the platform, whereas the second factor captured perceived pleasantness and enjoyment of the interaction experience. Factor loadings ranged from .675 to .884 for the ease-of-use dimension and from .711 to .954 for the pleasantness dimension, with generally low uniqueness values supporting the adequacy of the solution. Both subscales demonstrated good internal consistency. The ease-of-use factor showed α = .86 and ω = .86, while the pleasantness factor showed α = .87 and ω = .82, supporting the reliability of the questionnaire dimensions in the present sample.

**References**

Andresen, E. M., Malmgren, J. A., Carter, W. B., & Patrick, D. L. (1994). Screening for depression in well older adults: evaluation of. *Prev Med, 10*, 77-84.

Didino, D., Taran, E. A., Barysheva, G. A., & Casati, F. (2019). Psychometric evaluation of the Russian version of the flourishing scale in a sample of older adults living in Siberia. *Health and Quality of Life Outcomes, 17*(1), 34. <https://doi.org/10.1186/s12955-019-1100-6>

Diener, E., Wirtz, D., Tov, W., Kim-Prieto, C., Choi, D. W., Oishi, S., & Biswas-Diener, R. (2010). New well-being measures: Short scales to assess flourishing and positive and negative feelings. *Social Indicators Research, 97*(2), 143-156. <https://doi.org/10.1007/s11205-009-9493-y>

Flora, D. B. (2020). Your coefficient alpha is probably wrong, but which coefficient omega is right? A tutorial on using R to obtain better reliability estimates. Advances in Methods and Practices in *Psychological Science, 3*(4), 484-501. <https://doi.org/10.1177/2515245920951747>

Kenny, D. A., Kaniskan, B., & McCoach, D. B. (2015). The performance of RMSEA in models with small degrees of freedom. *Sociological methods & research, 44*(3), 486-507. <https://doi.org/10.1177/0049124114543236>

Kilburn, K., Prencipe, L., Hjelm, L., Peterman, A., Handa, S., & Palermo, T. (2018). Examination of performance of the Center for Epidemiologic Studies Depression Scale Short Form 10 among African youth in poor, rural households. *BMC psychiatry, 18*(1), 201. <https://doi.org/10.1186/s12888-018-1774-z>

Mohebbi, M., Nguyen, V., McNeil, J. J., Woods, R. L., Nelson, M. R., Shah, R. C., ... & ASPREE Investigator Group. (2018). Psychometric properties of a short form of the Center for Epidemiologic Studies Depression (CES-D-10) scale for screening depressive symptoms in healthy community dwelling older adults. *General hospital psychiatry, 51*, 118-125. <https://doi.org/10.1016/j.genhosppsych.2017.08.002>

Niu, L., He, J., Cheng, C., Yi, J., Wang, X., & Yao, S. (2021). Factor structure and measurement invariance of the Chinese version of the Center for Epidemiological Studies Depression (CES-D) scale among undergraduates and clinical patients. *BMC psychiatry, 21*(1), 463. <https://doi.org/10.1186/s12888-021-03474-x>

Padmanabhanunni, A., & Pretorius, T. B. (2023). Further refinement of the Center for Epidemiological Studies Depression Scale-10: complementary evidence From item response theory and classical test theory. *Psych, 6*(1), 21-33. <https://doi.org/10.3390/psych6010002>

Philippi, P., Baumeister, H., Apolinário-Hagen, J., Ebert, D. D., Hennemann, S., Kott, L., ... & Terhorst, Y. (2021). Acceptance towards digital health interventions–model validation and further development of the unified theory of acceptance and use of technology. Internet Interventions, 26, 100459. <https://doi.org/10.1016/j.invent.2021.100459>
